# Machine learning-based stratification of prediabetes and type 2 diabetes progression

**DOI:** 10.1186/s13098-025-01786-6

**Published:** 2025-06-18

**Authors:** Marwa Matboli, Abdelrahman Khaled, Manar Fouad Ahmed, Manar Yehia Ahmed, Radwa Khaled, Gena M. Elmakromy, Amani Mohamed Abdel Ghani, Marwa M. El-Shafei, Marwa Ramadan M. Abdelhalim, Asmaa Mohamed Abd El Gwad

**Affiliations:** 1 Department of Medical Biochemistry and Molecular Biology, Faculty of Medicine, Ain Shams University, Cairo, 11566 Egypt; 2 Bioinformatics Group, Center of Informatics Sciences (CIS), School of Information Technology and Computer Sciences, Nile University, Giza, Egypt; 3 Biotechnology Department, Faculty of Science, Cairo University, Cairo, 11566 Egypt; 4 Endocrinology & Diabetes Mellitus Unit, Department of Internal Medicine, Badr University in Cairo, Badr, Egypt; 5 Clinical Pathology, Faculty of Medicine, Ain Shams University, Cairo, 11566 Egypt; 6 Pathology Department, Faculty of Oral and Dental Medicine, Misr International University, Cairo, Egypt

**Keywords:** Diabetes mellitus, T2DM, Machine learning, Extra tree classifier, RNA

## Abstract

**Background:**

Diabetes mellitus, a global health concern with severe complications, demands early detection and precise staging for effective management. Machine learning approaches, combined with bioinformatics, offer promising avenues for enhancing diagnostic accuracy and identifying key biomarkers.

**Methods:**

This study employed a multi-class classification framework to classify patients across four health states: healthy, prediabetes, type 2 Diabetes Mellitus (T2DM) without complications, and T2DM with complications. Three models were developed using molecular markers, biochemical markers, and a combined model of both. Five machine learning classifiers were applied: Random Forest (RF), Extra Tree Classifier, Quadratic Discriminant Analysis, Naïve Bayes, and Light Gradient Boosting Machine. To improve the robustness and precision of the classification, Recursive Feature Elimination with Cross-Validation (RFECV) and a fivefold cross-validation were used. The multi-class classification approach enabled effective discrimination between the four diabetes stages.

**Results:**

The top contributing features identified for the combined model through RFECV included three molecular markers—miR342, NFKB1, and miR636—and two biochemical markers the albumin-to-creatinine ratio and HDLc, indicating their strong association with diabetes progression. The Extra Trees Classifier achieved the highest performance across all models, with an AUC value of 0.9985 (95% CI: [0.994–1.000]). This classifier outperformed other models, demonstrating its robustness and applicability for precise diabetes staging.

**Conclusion:**

These findings underscore the value of integrating machine learning with molecular and biochemical markers for the accurate classification of diabetes stages, supporting a potential shift toward more personalized diabetes management.

**Supplementary Information:**

The online version contains supplementary material available at 10.1186/s13098-025-01786-6.

## Introduction

Diabetes mellitus has always been a major worldwild concern, especially in low-income countries like Egypt, with continuously rising prevalence trends affecting the state’s healthcare and economy [1–3]. According to the “International Diabetes Federation” (IDF) latest version of the Diabetes Atlas, Egypt occupied the tenth position worldwide in 2021 in the number of adults that suffer from diabetes, with 10.9 million patients. It is also projected, according to the IDF Atlas, that Egypt will have 20 million patients in 2045, occupying ninth rank worldwide [4]. Long-standing diabetes can lead to several health problems like stroke, retinopathy, diabetic kidney disease and peripheral neuropathy [5]. Despite the huge burden of these disease-associated complications,early detection of prediabetics and diabetic patients can ameliorate these complications [6]. Highlighting the urgent need to find markers to diagnose prediabetics and diabetes in the early stages before complications occurs [7].

Recently, a huge number of molecular pathways have beenimplicated in the pathogenesis of diabetes mellitus type 2 (T2DM) & transition to a prediabetic state, among these pathways are insulin resistance, mammalian target of rapamycin (mTOR) [8] and autophagy [9]. Many papers have investigated the role of Insulin-Like Growth Factor 1 Receptor (IGF-1R) and the mTOR genes in insulin resistance and mTOR pathway in T2DM [10–12]. Also, mTOR [13], nuclear factor NF-kappa-B (NFKB1) [14] and RB1-inducible coiled-coil 1 (RB1CC1) [15] are autophagy-related genes that affect diabetes pathogenesis. Epigenetic modifiers such as miRNAs engage in diseases pathogenesis [16, 17]

Due to highly complicated interactions in disease pathogenesis, environmental factors, and endless mysteries of the genetic code, there has always been an emerging need for more advanced technologies to predict disease occurrence due to these factors [18]. Machine learning (ML) represented one of the best candidates in this field of study, as it learns from the natural code itself [19]. ML algorithms process big data acquired from previous cases, leading to prediction of future diabetic patients’ outcomes.

Recent ML models for T2DM stratification prioritize clinical parameters or single biomarkers, neglecting the interplay between molecular dysregulation (e.g., miR-342, NFKB1) and systemic metabolic dysfunction (e.g., albumin creatine ratio, HDLc) or focusing narrowly on single omics layers (genomic or proteomic) without integrating multi-dimensional biological data. This limits their ability to capture the heterogeneous pathophysiology underlying diabetes progression and complications. This limits their utility in identifying high-risk prediabetes or early T2DM subgroups [20, 21]. By integrating molecular and biochemical markers, our framework bridges this gap, leveraging miRNAs’ early predictive power [22]and biochemical indicators’ systemic relevance [23, 24] to enable precision staging. While longitudinal prediction remains a future goal, our cross-sectional stratification aligns with American Diabetic Association (ADA) recommendations for biomarker-based risk assessment [25]. Moreover, ML offers a better understanding of the complex genetic pathways leading to improvements in diagnosis, risk stratification, monitoring, personalized treatment and cost efficiency improvement [26, 27]. Recent studies have demonstrated the utility of machine learning for enhancing diabetes risk prediction through integration of genetic profiles and dynamic physiological data [28], as well as for predicting diabetes-related complications using ML frameworks [29]*.*

We aim to use integrated biochemical, molecular, and ML to identify potential biomarker panel for discriminating prediabetic, non-complicated T2DM, and complicated T2DM patients.

## Material and method

### Bioinformatic tool to retrieve the marker panel of the study

The biomarkers (mRNAs and miRNAs) were selected through a structured, multi-step integrated bioinformatics pipeline and previous literature validation studies designed to prioritize relevance to T2DM pathogenesis, functional annotations, and prior evidence of differential expression (supplementary Table S1).

**Fig. 1:**
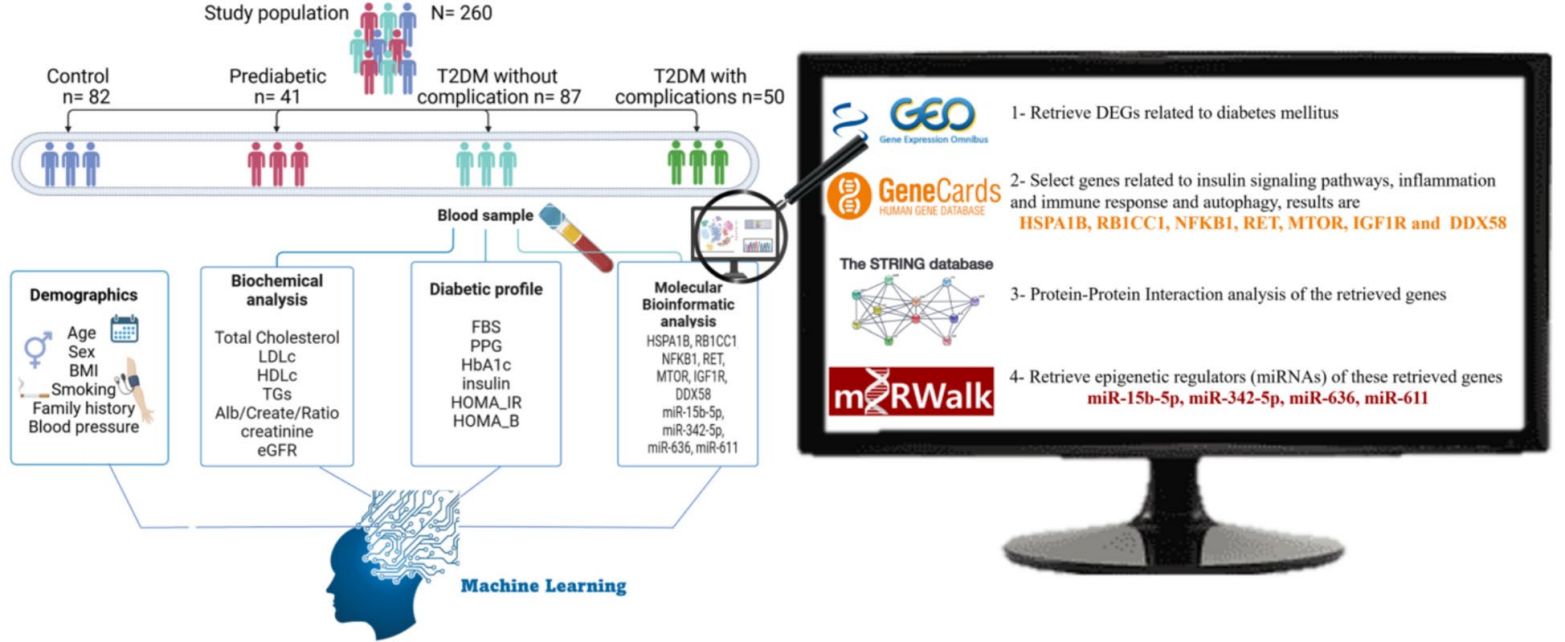
Blueprint of the study design

The Gene Expression Omnibus (GEO) database was used to retrieve mRNAs related to T2DM using specific keywords like “Type 2 Diabetes Mellitus”, and “Insulin Resistance” (https://www.ncbi.nlm.nih.gov/gds/, accessed in July 2024). The selection criteria included expression profiling tested by array, samples collected from both diabetic patients and normal samples, and datasets used for analysis consisting of more than five samples. Based on these criteria, we selected two datasets that contained differentially expressed genes (DEGs) (Supplementary Tables S2). The GeneCards database was used for gene ontology as we selected genes related to insulin signaling pathways, inflammation and immune response, and autophagy that are highly correlated with T2DM pathogenesis (https://www.genecards.org/, accessed in July 2024) (Fig. S1). The STRING database was used to explore protein-protein Interaction of the retrieved genes (https://string-db.org/, accessed in July 2024) (Fig. S2). So HSPA1B, RB1CC1, NFKB1, RET, MTOR, IGF1R and DDX58 mRNAs were chosen due to their previous differential expression in T2DM [30–33]. To identify the epigenetic regulators of these DEGs, we first choose miRNAs interacting with the selected DEGs using the mirWalk database (http://mirwalk.umm.uni-heidelberg.de/) (Fig S3). miR-15b-5p, miR-342-5p, miR-636, and miR-611 interact with retrieved DEG and are represented in Furthermore, A pairwise local sequence alignment between miRNA and mRNA was performed using the EMBOSS Water online tool (https://www.ebi.ac.uk/jdispatcher/psa/emboss_water) [34]. The miRNA sequence was retrieved from the miRDB database (https://mirdb.org/). Whereas for the mRNA sequence, it was retrieved from the Nucleotide database from NCBI (https://www.ncbi.nlm.nih.gov/nuccore/). As in supplementary table S3-Identity and similarity score for alignment between mRNA and miRNAs. Lastly, we chose to focus on the chosen molecular parameters, excluding the following: a) Genes/miRNAs with inconsistent expression across datasets. b) Biomarkers lacking functional annotations in T2DM pathways (e.g., non-inflammatory genes). Figure 1 demonstrates blue print of this research

### Subject of study and clinical parameters

This study included four groups with a total 260 subjects. The healthy group included 82 subjects, the prediabetic group had 41 subjects, then the without complications group had 87 patients, and finally, 50 patients were in the “T2DM with complications” group. Healthy group data were collected from regular checkups at hospitals of “Ain Shams University”. Healthy controls were selected to be without prior diabetic history, with normal glucose levels. For the other 3 groups classification, the “American Diabetes Association” classification was adopted. Glucose levels were examined for fasting and postprandial, along with glycated hemoglobin A1C. [35]. Then the diabetic group was subdivided into diabetic with complications and diabetic without complications groups. The Faculty of Medicine Research Ethical Committee FWA000017585/FAMSU P28/2022 at “Ain Shams University” approved this study. All participants in this study submitted written informed consent before participation and sample collection.

Clinicopathological info of the study population regarding sex, age, family history, smoking, and BMI was reported. Moreover, fasting glucose, postprandial, HbA1c, insulin, Homeostasis Model Assessment of Beta-cell function (HOMA-B) as an indicator of beta cell function was calculated as “20 × insulin in mIU/ml)/(glucose in mmol/L – 3.5” [36], Homeostasis Model Assessment of Insulin Resistance (HOMA-IR) as an indicator of insulin resistance was calculated as “Fasting insulin (μU/L) x fasting glucose (nmol/L)/22.5” [37], total cholesterol, LDLc, HDLc, TGs, Alb/Create/Ratio, creatinine and eGFR were examined using a multifunctional biochemistry analyzer (AU680, Beckman Coulter Inc., Kraemer Blvd., Brea, CA 92821,USA). Collected blood samples were processed for sera collection, then sera was kept at −80C for further processing.

The “miRNEasy extraction kit” that is produced by (Qiagen, Hilden, Germany) was used for purification of RNA from samples. Then validation of the quality and the purity of purified RNA was done using the “Qubit 3.0 Fluorimeter” (Invitrogen, Life Technologies, Malaysia) and “Qubit TM ds DNA HS and RNA HS Assay Kits” (Cat. no. Q32851, Q32852). Finally, purified RNA was reverse transcripted by the “miScript II RT kit” by Qiagen, and the process was performed in the “Rotor-Gene Thermal Cycler” (Thermo Electron Waltham, MA).

Differential expression assessments for RET, IGF1R, mTOR, HSPA1B, DDX, NFKB1, and RB1CC1 mRNAs were done using “Quanti-tect SYBR Green Master Mix, Cat No. 204143” by Qiagen and Quanti-Tect Primer Assays as in Supplementary Table S4 using GAPDH as an endogenous control as per the manufacturer’s directions. On the other hand, differential expression assessments of miR 342, miR636, miR 15b, and miR611 were done using the “miScript SYBR Green PCR Kit” GeneGlobeID as in Supplementary Table S4 Cat. No. 339306, by Qiagen and miScript LNA primer assays, while using SNORD72 as an endogenous control as per the manufacturer’s directions. Each test was done twice. The Leviak method where RQ = 2 ^−∆∆Ct^. was adopted for RNA signature relative expression calculation. This research used “Applied Biosystems 7500 v2.3” software to analyze results and to calculate samples'CT values. We used suitable standardization strategies according to MIQE guidelines to figure out any error at any stage along experimental processes.

### Statistical analysis

We used SPSS version 25 (IBM, Chicago, USA) for statistical analysis. For categorical data expression, the study utilized number and precent, while for quantitative medians and interquartile range were used. The chi-square test was used for assessment of the categorical clinicodemographic. For analysis of continuous data Mann–Whitney and Kruskal–Wallis tests were used for comparing two or more groups, respectively. The Shapiro–Wilk test was employed for variables’ normality. Dunn's multiple comparison tests after Kruskal–Wallis test were employed for study group comparison. Ap-value of 0.05 or less was used for statistical significance reporting.

### Machine learning models

One of the primary objectives of this study was to develop a predictive model that can accurately classify individuals into four distinct health categories: healthy, prediabetes, T2DM without complications, and T2DM with complications, using both molecular and biochemical markers (Table 1). By analyzing and comparing these data types, the study aimed to identify key biomarkers that can discriminate between these disease stages and offer clues about the progression of Type 2 Diabetes Mellitus (T2DM) and to enhance the predictive accuracy, enabling earlier and more precise categorization of individuals along the diabetes spectrum. This project sought to develop models capable of supporting clinical decision-making, potentially facilitating more personalized monitoring and intervention strategies for individuals at different stages of T2DM.

**Table 1:** Molecular, and biochemical features used in ML models

Molecular (11 features)	Biochemical (14 features)
1. miR 342 2. miR636 3. miR 15b 4. miR611 5. RET 6. IGF1R 7. mTOR 8. HSPA1B 9. DDX 10. NFKB1 11. RB1CC1	1. Fasting blood Glucose 2. Postprandial blood Glucose 3. HbA1c 4. Insulin 5. HOMA IR 6. Total Cholesterol 7. Systolic blood pressure 8. Diastolic blood pressure 9. LDLc 10. HDLc 11. TGs 12. Albumin Creatinine Ratio 13. Creatinine 14. eGFR

Our dataset included 260 human samples, divided into four distinct groups based on health status: healthy (82 samples), prediabetes (41 samples), T2DM without complications (87 samples), and T2DM with complications (50 samples). The target variable in this study, representing the four distinct stages of diabetes, was encoded to allow effective multi-class classification in the machine learning framework. Specifically, each category was assigned a numerical label: healthy (0), prediabetes (1), T2DM without complications (2), and T2DM with complications (3). This labeling approach did not impose any ordinal relationship between classes but rather treated each state as a distinct, categorical class. This encoding allowed the machine learning models to distinguish between discrete health conditions, enabling accurate multi-class predictions that capture the progression of T2DM across different stages.

**Fig. 2:**
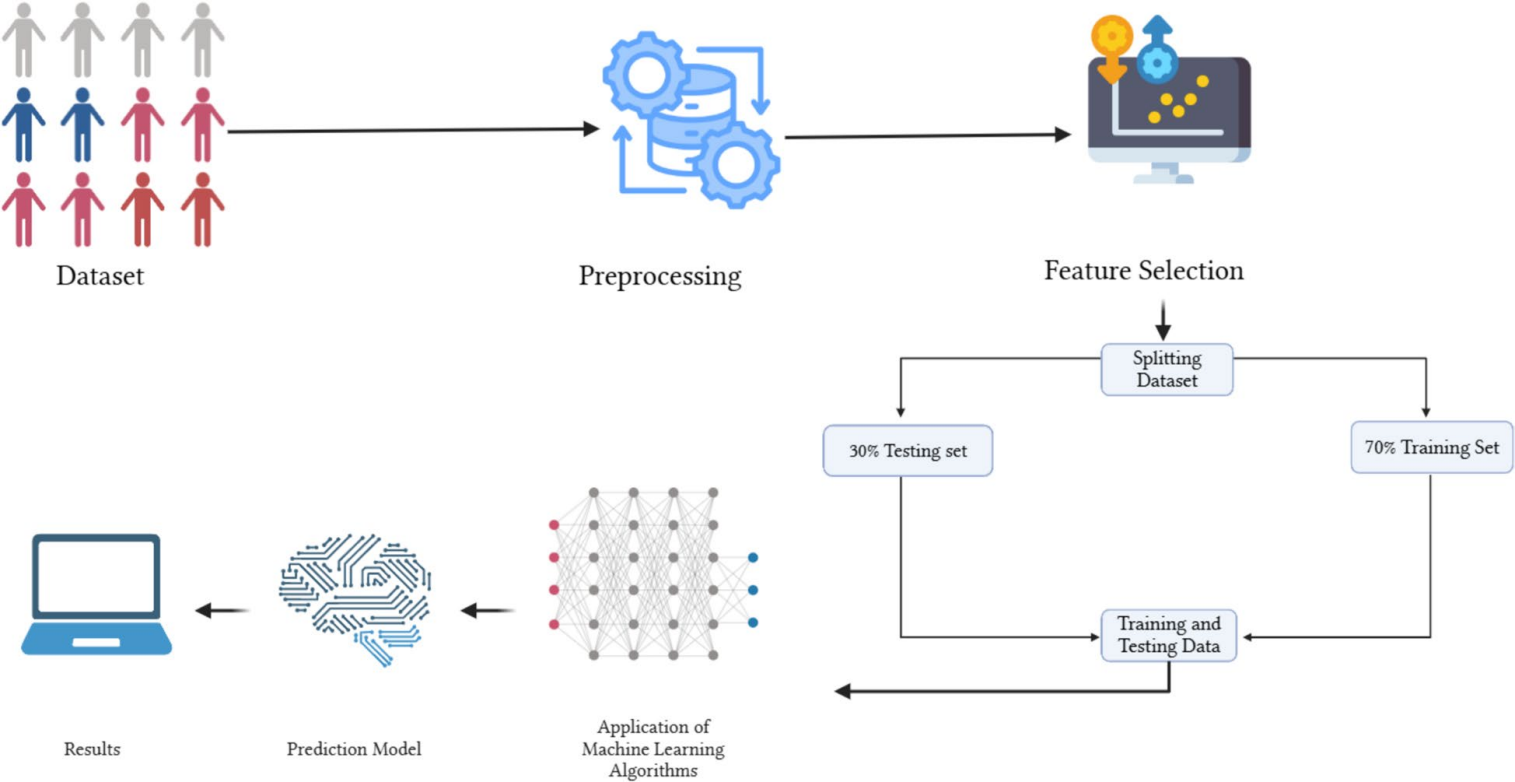
Summary of the machine learning Workflow

The dataset was divided into a 70/30 split for training and testing, ensuring that a representative sample of each category was included in both sets. During model development, we created three distinct models to assess feature contributions (Table 2): one model using only molecular features, a second model using only biochemical features, and a final combined model integrating all features. We tested five classifiers: Naive Bayes, Random Forest (RF) Classifier, Quadratic Discriminant Analysis, Extra Trees Classifier, and LightGBM. Figure 2 summarizes the machine learning workflow.

**Table 2:** The three predictive models were applied to the five classifiers

Model	Data type
1	Molecular
2	Biochemical
3	Molecular + Biochemical

### Dataset preprocessing (remove null/outliers)

Data preprocessing is a vital phase, especially for healthcare-related datasets, where missing values and other imperfections can compromise data quality [38]. Improving the dataset's usability and reliability after collection is essential, and data preprocessing fulfills this need. It is key to applying machine learning techniques successfully, as it ensures accurate outcomes and reliable predictions. A core part of preprocessing involves addressing missing values, such as excluding entries where values are zero, as these are typically unrealistic in healthcare scenarios. Removing irrelevant features or instances allows us to form a streamlined feature subset, known as feature subset selection, which minimizes data dimensionality and boosts computational efficiency. Thus, we analyzed the dataset to assess for any missing data; however, upon examination, we found that no missing data was present.

Outliers represent unusual patterns in data that fall outside the typical range of behavior. Identifying these anomalous patterns, or error outliers, is important for managing them effectively to improve prediction accuracy, particularly in machine learning models. If an outlier is deemed an error, it can simply be removed from the dataset [39, 40]. A commonly used method to detect outliers in a dataset is the interquartile range (IQR). The interquartile range (IQR) was calculated for each numeric attribute in the dataset, filtering out instances that fell outside a defined range to aid in identifying and removing outliers. This process helped improve the dataset's integrity, reducing the potential influence of outliers on subsequent analysis or modeling results.

### Correlation analysis

To explore the relationships among different features, we performed a correlation analysis. The resulting correlation matrix illustrates the correlation coefficients for each variable pair, with values ranging from −1 to 1. Positive coefficients signify a direct relationship, while negative coefficients reflect an inverse relationship. The strength of the relationship is indicated by the magnitude of the correlation coefficient, where values near 1 or −1 indicate stronger correlations. This analysis provided valuable insights into the interactions between biomarkers, guiding our feature selection for subsequent modeling [41].

### Normalization

Data normalization is essential for enhancing the performance of machine learning algorithms. It helps mitigate bias from features with larger numerical values, allowing for fair weighting of each variable during training. Normalization also improves numerical stability, reduces training duration, and enables meaningful comparisons among features. Since some continuous attributes in the dataset span a broad range of values, they can significantly affect classifier performance. To scale continuous features to a [0,1] range, min–max normalization is applied [42].

### Synthetic minority over-sampling technique (SMOTE)

The combined dataset used in this research consists of 87 samples for T2DM without complications, 82 for healthy individuals, 50 for T2DM with complications, and 41 for prediabetes, reflecting an imbalance across class distributions, which may have led to reduced predictive performance for the minority class in the model [43]. To address this imbalance during training, the Synthetic Minority Oversampling Technique (SMOTE) was applied for the training data set, while the testing data set remained unaltered [44]. Each class was up sampled to 87 samples to ensure balanced representation during training. SMOTE was applied within the cross-validation folds, ensuring no data leakage and preserving the validity of model evaluation.

SMOTE was used in two separate phases, each implemented with proper isolation. First, during feature selection with RFECV using RandomForestClassifier, SMOTE was applied within a scikit-learn pipeline inside each cross-validation fold, oversampling only the training partition in each fold. After selecting the top features, we used PyCaret for final prediction and evaluation. PyCaret's internal pipeline applied SMOTE again (via fix_imbalance = True) within each cross-validation fold, independently and freshly from the previous phase, again limited to the training subsets. At no point was SMOTE applied before the train/test split, and synthetic samples never leaked into the testing data. This two-step approach ensured unbiased feature selection and model evaluation while maintaining class balance throughout.

### Feature selection

Feature selection was applied to streamline the data, reducing both its complexity and size, which improved the model's learning efficiency. By choosing only the most relevant features, this approach accelerated the model and enhanced its precision, ultimately boosting predictive performance by minimizing noise.

### Recursive feature elimination with cross-validation (RFECV)

RFECV is a feature selection method that employs a machine learning algorithm to identify the most relevant features for the detection task. To enhance robustness, RFECV integrates recursive feature elimination with cross-validation, allowing it to determine the optimal set of features that maximizes model performance [45].

RFECV employs a classification model to evaluate each feature’s significance, iteratively removing those that do not improve classification accuracy. This backward selection process begins with the full set of features, gradually eliminating less impactful ones, and ultimately identifies the most effective subset for classification. In this study, RFECV was implemented using a RandomForestClassifier model as the estimator, with cross-validation set to fivefold StratifiedKFold. This approach enabled us to systematically remove less significant features, producing a streamlined and efficient model with an optimal balance between simplicity and predictive accuracy.

Throughout the recursive elimination process, the accuracy metric is assessed at each iteration to evaluate the effect of feature removal on model performance. By monitoring the changes in accuracy metrics with each iteration, we can gain insights into the significance and contribution of each feature to the model's effectiveness. The optimal set of features was identified based on the classifier that achieved the highest overall accuracy.

### Feature importance

Feature importance analysis is critical for identifying the most influential factors in diabetes prediction. In this study, we employ comprehensive feature importance techniques to gain valuable insights. Using the RFalgorithm, we rank features based on their contribution to prediction accuracy, providing a clear hierarchy of significance. To enhance interpretability, we incorporate visualizations such as bar plots, which display the relative importance of each feature and facilitate the identification of key predictors. These methods collectively allow us to better understand the dominant variables impacting the model’s performance.

### Cross-validation

K-fold Cross-Validation (k-CV) is a statistical method employed to assess and compare the performance of classifiers in machine learning algorithms. It divides the dataset into two segments: one for training the model and the other for validation or testing. In k-CV, the data is partitioned into k equal (or nearly equal) segments or folds. Subsequently, k iterations of training and validation are conducted, with each iteration utilizing a different fold for validation while the remaining k-1 folds are used for training [46, 47].

In our research, we employed fivefold cross-validation to assess the effectiveness of our machine learning models. We utilized stratified Kfold cross-validation to ensure that each fold preserved the same class distribution as the entire dataset, thereby reducing potential biases and increasing the reliability of our findings. The dataset was divided into five subsets, each reflecting a representative distribution of the target variable classes. For every fold, we trained the model using four of the subsets and validated it on the remaining one. This procedure was repeated five times, ensuring that each subset was utilized for validation once. By implementing stratified K-fold cross-validation, we aimed to rigorously evaluate our model on unseen data during each fold, thus enhancing the accuracy and reproducibility of our results.

### Measures to mitigate overfitting

To minimize the risk of overfitting and ensure the robustness of our model, we implemented several deliberate strategies throughout the analysis pipeline. These included rigorous data cleaning and confirm the absence of missing values, also using the MinMax scaler for normalization to harmonize feature scales and prevent any single variable from disproportionately influencing the model. During feature selection, we employed Recursive Feature Elimination with Cross-Validation (RFECV), a method that iteratively removes less informative features based on cross-validation performance, thereby reducing noise and model complexity. SMOTE was applied strictly within the training folds during both the feature selection and classification phases to address class imbalance without introducing data leakage. Furthermore, we utilized repeated cross-validation to evaluate model generalization across multiple data splits. Together, these measures were systematically applied to enhance the generalizability and reliability of our machine learning models.

### Machine learning algorithms for classification

#### Extra tree classifier

The Extra Trees classifier, also known as the"Extremely Randomized Trees"classifier, is a bagging-based machine learning algorithm that builds multiple decorrelated decision trees (DT) using random samples from the training dataset. In machine learning, both Extra Trees classifiers and regressors contribute to constructing a collection of trees aimed at reducing overfitting and enhancing classification accuracy [48].

### Random forest

Random Forest (RF) is a classification technique that utilizes multiple decision trees, originally proposed by Breiman [49]. RF is a versatile machine learning method, capable of performing both classification and regression tasks. It is based on bagging and plays a key role in ensemble machine learning approaches [50]. RF has been widely applied in biomedical research.

Unlike a single DT algorithm, RF constructs a large ensemble of trees. When predicting the class of a new sample, each tree in the RF provides its classification result, effectively"voting,"and the overall prediction is determined by the majority vote across all trees. For regression tasks, the RF output is the mean of the predictions from each individual tree [51].

### Naïve bayes

Naive Bayes is a machine learning algorithm commonly used for classification tasks. It is based on Bayes'theorem and assumes that features are conditionally independent once the class label is known. This assumption allows the algorithm to be fast and scalable to high-dimensional datasets. For classification applications, especially in text categorization and spam filtering, Naive Bayes is a simple yet effective method. It is resilient to irrelevant features and capable of handling missing data efficiently [52].

### Light gradient boosting machine

LightGBM (LGBM) is regarded as a high-performance gradient boosting (GB) framework built upon the DT algorithm [53]. It is commonly applied in tasks such as classification and ranking, utilizing a leaf-wise splitting approach for optimal fit. Data improvement techniques can evaluate its performance, specifically by calculating the variance after partitioning [54].

### Quadratic discriminant analysis

Quadratic Discriminant Analysis (QDA) is a more advanced version of Linear Discriminant Analysis (LDA) that enables non-linear separation of data by accounting for class-specific covariances. While both QDA and LDA function as classifiers and dimensionality reduction techniques, QDA provides greater flexibility in handling data with complex boundaries by allowing each class to have its own covariance structure, unlike the linear assumption in LDA [55].

### Evaluation

In the evaluation phase of our machine learning project, we employed a comprehensive set of metrics to thoroughly assess model performance, including accuracy, recall, precision, F1-score, Matthew’s correlation coefficient (MCC), and area under the curve (AUC).

Accuracy measurement serves as a key metric for assessing the performance of a classification model. It calculates the ratio of correctly classified instances to the total instances. This metric is determined by dividing the count of accurate predictions by the total number of predictions generated [56]

Recall, also referred to as sensitivity or the true positive rate, indicates the proportion of actual positive instances that are accurately identified as positive. It is calculated as the ratio of true positives to the total of true positives and false negatives. Recall emphasizes reducing the number of false negatives.

Precision indicates the proportion of predicted positive instances that are truly positive. It is determined by the ratio of true positives to the total of true positives and false positives. Precision aims to reduce the incidence of false positives.

The F1-score is a metric that integrates precision and recall into a unified score, offering a balanced assessment of both. It is especially valuable for imbalanced datasets, as it weighs both precision—focused on reducing false positives—and recall—focused on reducing false negatives. Together, these metrics provide a comprehensive evaluation, enhancing the effectiveness of machine learning models across different applications [57].

The Matthews correlation coefficient (MCC) is a metric commonly applied to evaluate the quality of both binary and multiclass classification models. It is frequently used in machine learning and bioinformatics, particularly for assessing models on imbalanced datasets or when class sizes vary significantly. The MCC score ranges from −1 to + 1, where + 1 signifies a perfect prediction, 0 reflects a random prediction, and −1 represents an entirely incorrect prediction [58].

the receiver operating characteristic (ROC) curve and area under the curve (AUC) metric to evaluate the model's discrimination ability between classes. The AUC, along with the ROC curve, helped visualize the trade-offs between true positive and false positive rates at various thresholds, adding depth to our evaluation of model performance.

Model Reproducibility and Hyperparameter Settings.

To ensure reproducibility, we consistently used session_id = 123 in PyCaret library and random_state = 44 parameters across all models. This guarantees consistent results across multiple runs.

The final combined model was built using ExtraTreesClassifier, the best-performing classifier identified by PyCaret. We used the following default hyperparameters provided by PyCaret:

- n_estimators = 100
- max_depth = None
- max_features ='sqrt'
- min_samples_split = 2
- min_samples_leaf = 1

## Packages

This study’s data processing was conducted in Python 3.7, leveraging several Python-based libraries to streamline the processing pipeline. The ‘pandas’ package (version 1.3.5) and ‘NumPy’ (version 1.20.3) were used for efficient data manipulation and analysis. For data visualization, ‘Seaborn’ (version 0.13.2) was employed to enhance graphical capabilities, while ‘Matplotlib.pyplot’ (version 3.5.0) provided a flexible toolkit for creating static, interactive, and animated visualizations. Machine learning tasks were primarily handled by Pycaret and scikit-learn (version 1.0.2), with ‘MinMaxScaler’ from ‘sklearn.preprocessing’ used for normalizing data, and ‘SMOTE’ from ‘imblearn.over_sampling’ applied to address class imbalance.

## Results

### Demographic and clinical data of four studied groups

This study was conducted among 260 study subjects, divided into 137 patients with T2DM, which was subdivided into 87 without complications and 50 with complications, 41 were prediabetics and 82 were healthy volunteers. Statistical analysis showed no significant differences regarding sex or age among the four studied groups (p > 0.05). However, there were significant differences when comparing smoking.

Family history, postprandial blood glucose, HbA1c, insulin, HOMA-IR, BMI, total cholesterol, TGs, and eGFR as we go from healthy control to prediabetic to complicated T2DM reach the largest levels in complicated T2DM patients (p < 0.05). Also, there were significant differences regarding systolic blood pressure, HDLc, and creatinine when the transition from prediabetic to non-complicated T2DM reached the highest level in complicated T2DM. Also, there were significant differences regarding diastolic Blood pressure and albumin/creatinine ratio when comparing healthy controls versus prediabetics and diabetics versus complicated diabetics. Finally, regarding fasting blood Glucose, HOMA_B, and LDLc, there was statistically significant difference when comparing healthy controls versus prediabetic and prediabetic versus T2DM patients. As for disease duration, there was a significant statistical difference between complicated and non-complicated T2DM groups, as in Table 3. (p < 0.05).

**Table 3:** Demographic and clinical data of four studied groups

	Healthy n = 82	Prediabetes n = 41	T2DM without complications n = 87	T2DM with complications n = 50	p-value
Age	54 (48–60)	52 (49–58)	53 (48–60)	53 (50–57)	0.732
Sex					0.732
Male	35 (42.7%)	18 (43.9%)	34 (39.1%)	17 (34%)	
Female	47 (57.3%)	23 (56.1%)	53 (60.9%)	33 (66%)	
Smoking					< 0.001
X smoker	1 (1.2%)	4 (9.8%)	6 (6.9%)	0 (0%)	
Negative	27 (32.9%)	9 (22%)	39 (44.8%)	50 (100%)	
Positive	54 (65.9%)	28 (68.3%)	42 (48.3%)	0 (0%)	
Family history					< 0.001
Positive	37 (45.1%)	33(80.5%)	67 (77%)	30 (60%)	
Negative	45 (54.9%)	8(19.5%)	20 (23%)	20 (40%)	
Duration of diabetes			8 (5–14)	96 (80.5–119)	< 0.001҂
Fasting blood glucose	77 (70–84.3)	120 (110–126)^a^	161 (130–206)^b,d^	191 (128–244)^c,e^	< 0.001
Postprandial blood glucose	120 (100–133)	150 (127–169)^a^	240 (181–320)^b,d^	371 (261–409)^c,e,f^	< 0.001
HbA1c	5.1 (5–5.6)	6.3 (6–6.4)^a^	9 (8–11)^b,d^	10.8 (9–12)^c,e,f^	< 0.001
Insulin	6 (4–7)	9.4 (8–10)^a^	16 (13–18)^b,d^	18 (15–21)^c,e,f^	< 0.001
HOMA_IR	0.601 (0.355–0.906)	2.56 (1.52–3.44)^a^	6.83 (5.2–8.61)^b,d^	10.6 (6.96–12.6)^c,e,f^	< 0.001
HOMA_B	140 (120–160)	100 (92–102)^a^	52 (45.5–66)^b,d^	52 (44–60)^c,e^	< 0.001
Systolic blood pressure	130 (120–140)	130 (120–140)	166 (120–188)^b,d^	177 (166–190)^c,e,f^	< 0.001
Diastolic blood pressure	80 (70–80)	110 (90–120)^a^	109 (99.5–118)^b^	128 (115–140)^c,e,f^	< 0.001
BMI	25 (23–29.8)	30 (29–33.5)^a^	35 (30–39)^b,d^	38 (33.4–40)^c,e,f^	< 0.001
Total Cholesterol	110 (90–132)	234 (226–270)^a^	315 (270–343)^b,d^	366 (320–400)^c,e,f^	< 0.001
LDLc	75 (66–90)	180 (166–190)^a^	230 (210–266)^b,d^	217 (190–266)^c,e^	< 0.001
HDLc	65 (60–69.8)	44 (39–49)^a^	29 (25–33.5)^b,d^	22 (19–25.8)^c,e,f^	< 0.001
TGs	109 (99–142)	75 (65–200)	200 (114–265)^b,d^	290 (210–324)^c,e,f^	< 0.001
Alb/Creat/Ratio	14 (11–20)	22 (20–28)^a^	25 (21.5–28)^b^	266 (233–289)^c,e,f^	< 0.001
Creatinine	0.8 (0.75–0.86)	0.8 (0.75–0.8)	1.1 (0.8–1.96)^b,d^	2 (1.7–2.58)^c,e,f^	< 0.001
eGFR	100 (95–107)	94 (90–97)^a^	44 (37.5–56)^b,d^	37.5 (32.3–40)^c,e,f^	< 0.001

HbA1c: “Hemoglobin A1c”, HOMA-IR: “Homeostatic Model Assessment for Insulin Resistance”, HOMA-B: “Homeostatic Model Assessment for Beta-cell Function”, BMI: “Body Mass Index”, HDLc: “High-Density Lipoprotein Cholesterol”, LDLc: “Low-Density Lipoprotein Cholesterol”, TGs: “triglycerides”, Alb/Creat/Ratio: “Albumin-creatinine ratio”, eGFR: “Estimated glomerular filtration rate”, Continuous data are presented as medians and interquartile range while categorical data are expressed as number & percentages. The Kruskal–Wallis test was used, when it was significated, it followed by Dunn's test for multiple comparison with reported significance (a, b, c, d, e, & f), where each letter denotes following comparisons a: Healthy versus prediabetes, b: Healthy versus T2DM without complications, c: Healthy versus T2DM with complications, d: prediabetes versus T2DM without complications, e: prediabetes versus T2DM with complications, f: T2DM without complications versus T2DM with complications. ҂Mann Whitney U test used for compering two groups only. The chi-square test is used to examine categorical variables

### The RNAs’ differential expression among the four studied groups

The expression levels of the RNA signature (miR-15b-5p/miR-342-5p/miR-636/miR-611/NFKB1/MTOR/IGF1R/RET/RB1CC1/HSPA1B/DDX58 mRNAs) were assessed in serum samples of the four study groups.

**Fig. 3:**
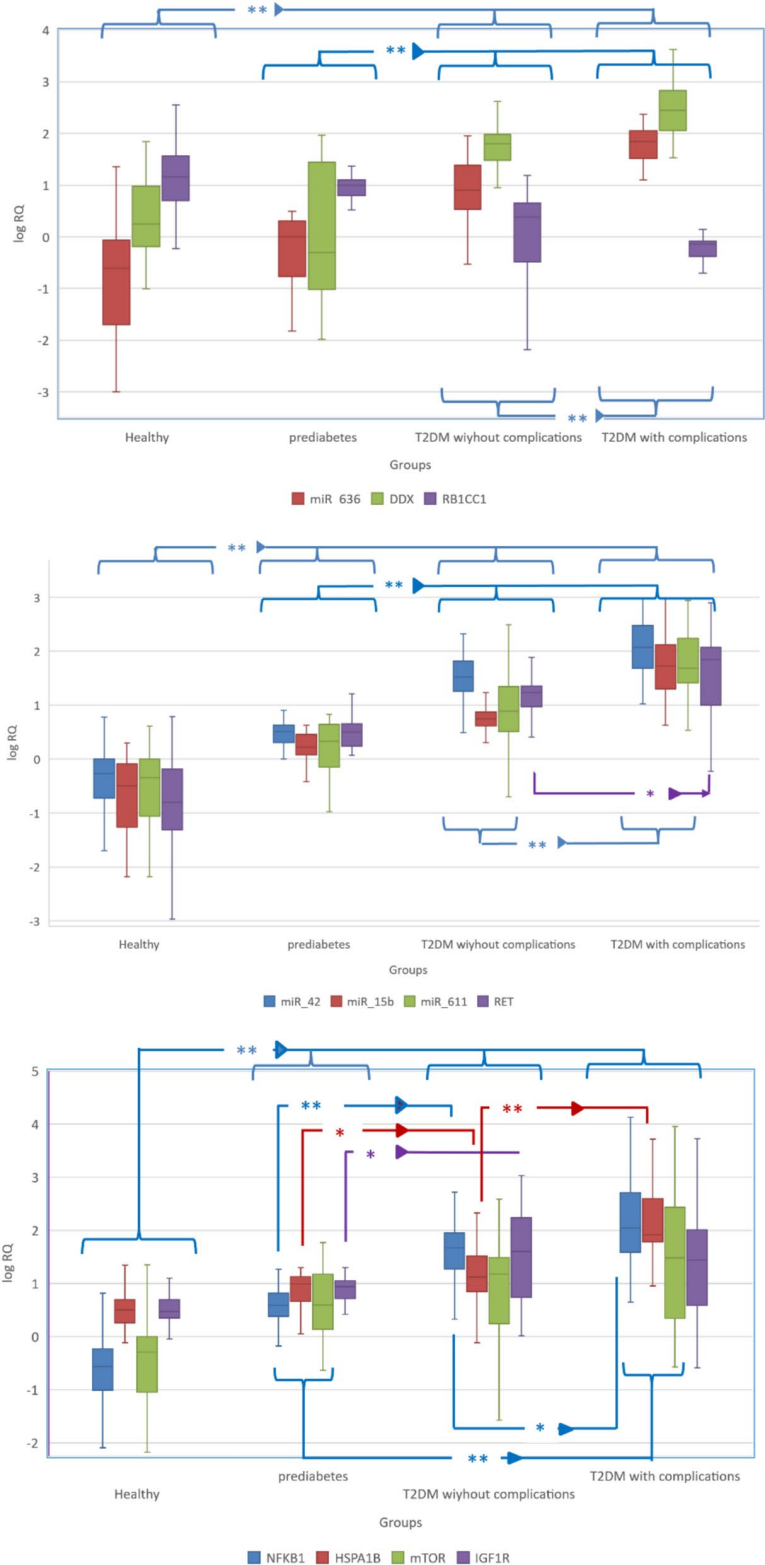
RNA panel differential expression across the four studied groups

On analysis of the results, during comparing prediabetic versus healthy control there was a significant increase in miR-15b-5p/miR-342-5p/miR-611/NFKB1/MTOR/IGF1R/RET/HSPA1B mRNAs levels. Also when prediabetic versus non complicated T2DM miR-15b-5p/miR-342-5p,/miR611/miR636/NFKB1/IGF1R/RET/HSPA1B and DDX mRNA levels were significantly increased however RB1CC1 mRNA was significantly decreased. Also, when comparing complicated versus non-complicated T2DM groups miR-15b-5p/miR-342-5p/miR 611, miRNA 636/NFKB1/RET/HSPA1B and DDX mRNA levels were significantly increased; however, RB1CC1 mRNA was significantly declined, as in Fig. 3 and Table 4.

**Table 4:** The RNA panel differential expression among the four studied groups

	Healthy n = 82	Prediabetes n = 41	T2DM without complications n = 87	T2DM with complications n = 50	p-value
miR_342	0.541 (0.19–1)	3.2 (2.1–4.17)	33.3 (18.3–66)^b,d^	117 (50.8–297)^c,e,f^	< 0.001
miR_636	0.249 (0.02–0.859)	1 (0.17–2.01)	8.02 (3.42–23.2)^b,d^	69 (34.9–108)^c,e,f^	< 0.001
miR_15b-5p	0.318 (0.0558–0.78)	1.67 (1.24–2.85)	5.55 (4.17–7.37)^b,d^	53.3 (20.8–131)^c,e,f^	< 0.001
miR_611	0.453 (0.090–1)	2.13 (0.841–4.32)	7.8 (3.35–21.1)^b,d^	48.1 (26.9–162)^c,e,f^	< 0.001
RET	0.159 (0.052–0.625)	3.16 (1.89–4.4)^a^	17 (9.77–22.5)^b,d^	69.6 (13.2–116)^c,e,f^	< 0.001
IGF1R	2.98 (2.28–4.86)	8.63 (5.24–10.9)^a^	39.7 (5.95–162)^d^	27.5 (4.16–79.7)^c,e^	< 0.001
mTOR	0.513 (0.0928–1)	3.94 (1.66–14.1)^a^	15 (1.78–28.9)^b,d^	30.2 (2.45–246)^c,e,f^	< 0.001
HSPA1B	3.17 (1.89–4.92)	9.78 (4.76–13.5)	13.2 (7.1–32)^b,d^	82.1 (62–327)^c,e,f^	< 0.001
DDX58	1.79 (0.674–8.88)	0.493 (0.0981–27.1)	64 (30.5–94)^b,d^	280 (120–646)^c,e,f^	< 0.001
NFKB1	0.273 (0.102–0.578)	3.89 (2.45–5.86)^a^	46.8 (18.8–89.5)^b,d^	110 (39.7–422)^c,e,f^	< 0.001
RB1CC1	14.5 (5.11–35.5)	9.83 (7.57–11.7)	2.39 (0.343–4.36)^b,d^	0.715 (0.425–0.8)^c,e,f^	< 0.001

RET: “Proto-oncogene receptor tyrosine kinase”, IGF1R: “Insulin-like Growth Factor 1Receptor”, mTOR: “mammalian target of rapamycin”, HSPA1B: “Hsp70 family Chaperones”, DDX58: “Retinoic acid-inducible gene-I”, NFKB1: “Nuclear factor NF-kappa-B”, RB1CC1: “RB1-inducible coiled-coil 1”, Data are presented as medians and interquartile range. The Kruskal–Wallis test was used, when it was significated, it followed by Dunn's test for multiple comparison with reported significance (a, b, c, d, e, & f), where each letter denotes following comparisons a: Healthy versus prediabetes, b: Healthy versus T2DM without complications, c: Healthy versus T2DM with complications, d: prediabetes versus T2DM without complications, e: prediabetes versus T2DM with complications, f: T2DM without complicationsversus T2DM with complications

### Correlation matrix analysis

**Fig. 4:**
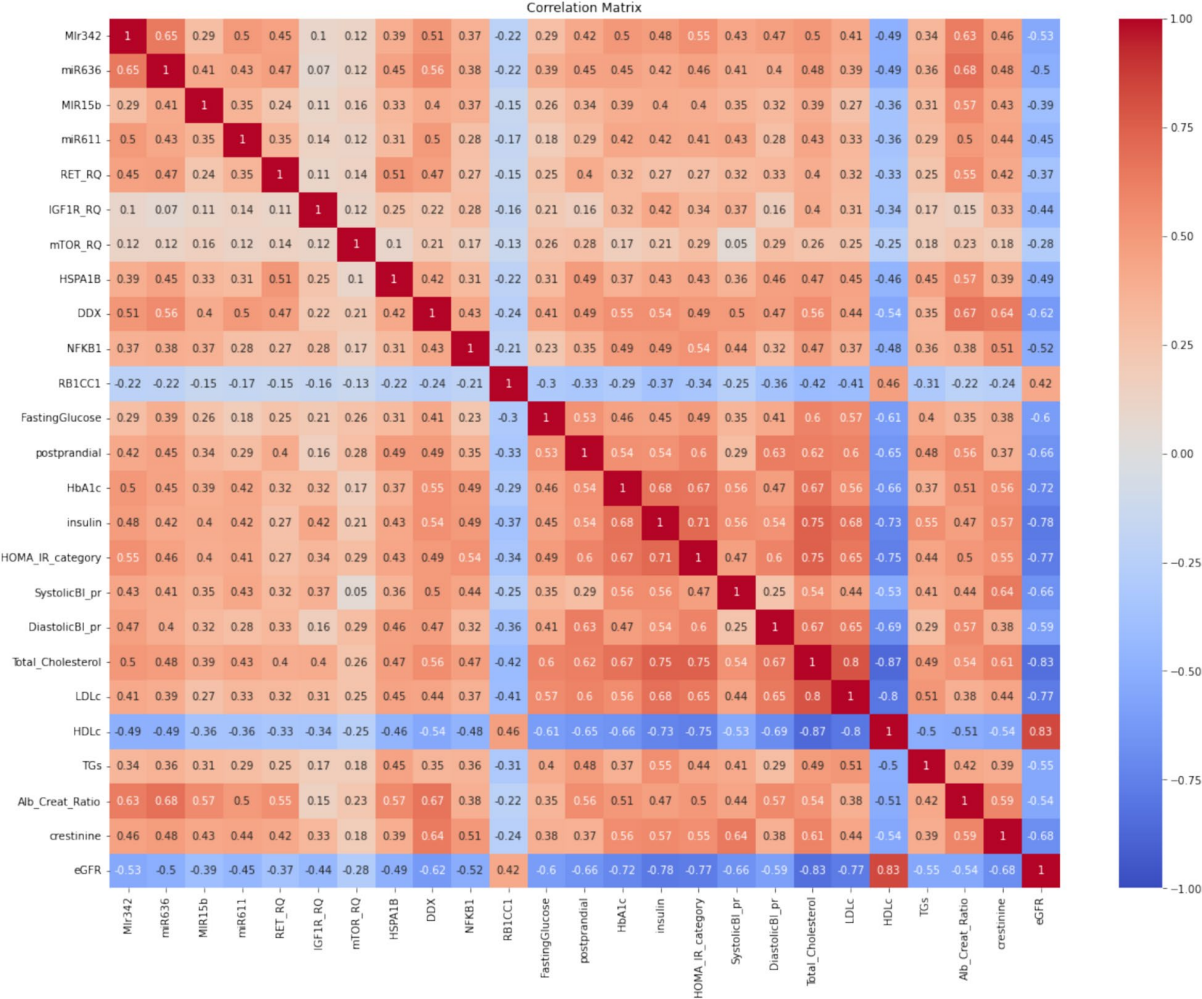
Show the correlation heatmap of T2DM dataset features

The correlation matrix shown in Fig. 4 illustrates the degree of correlation between pairs of features throughout the dataset, providing valuable insights into their interrelationships. Each cell within the matrix represents the calculated correlation coefficient for the respective feature pairs.

### Feature selection using RFECV-based random forest for T2DM prediction

One of the aims of this study is to utilize advanced machine learning methodologies to effectively predict the stages of T2DM. By examining biochemical and molecular markers, we strive to uncover significant biomarkers related to the disease.

**Fig. 5:**
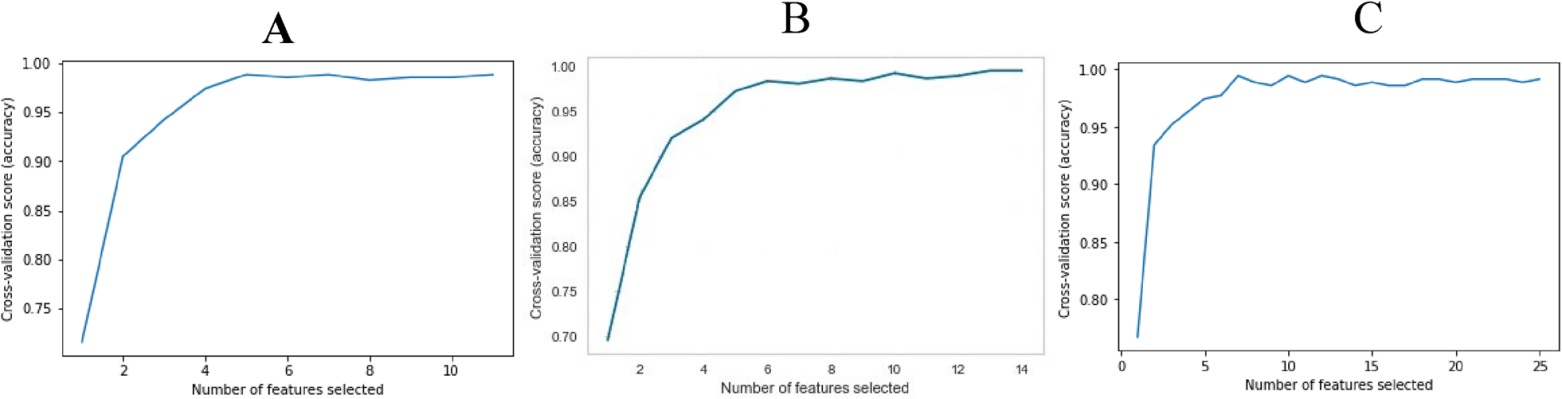
Feature Selection Performance Using RFECV. (**A**) Molecular, (**B**) Biochemical, (**C**) Combined set

In our analysis, we applied Recursive Feature Elimination with Cross-Validation (RFECV) to identify the most influential predictors for diabetes classification. The outcomes of the RFECV analysis and the accuracy metrics on the test set for each feature group are illustrated in Fig. 5.

RFECV successfully selected 5 out of 11 features for the molecular model, all 14 features from the biochemical model, and 7 out of 25 features for the combined model, while maintaining comparable levels of prediction accuracy. The selected and unselected features, are shown in.

(Table 5). These results highlight the importance of biomarkers chosen in predicting the progression of T2DM.

**Table 5:** Show the included and excluded features for each feature group

Model	Included Features	Excluded Features
Molecular Included: 5 Excluded: 6 Total: 11	miR 342 miR636 miR15b RET NFKB1	RB1CC1 miR611 IGF1R mTOR HSPA1B DDX
Biochemical Included: 14 Excluded: 0 Total: 14	Fasting Glucose Postprandial HbA1c Insulin HOMA-IR Total Cholesterol Systolic blood pressure Diastolic blood pressure LDLc HDLc TGs Albumin Creatinine Ratio Creatinine eGFR	None
Combined Included: 7 Excluded: 18 Total: 25	miR 342 miR636 miR 15b NFKB1 FastingGlucose HDLc Albumin Creatinine Ratio	Postprandial HbA1c Insulin HOMA IR Total Cholesterol Systolic blood pressure Diastolic blood pressure LDLc TGs Crestinine eGFR RET_RQ RB1CC1 miR611 IGF1R_RQ mTOR_RQ HSPA1B DDX

### Feature importance using RFECV-based random forest for T2DM prediction

**Fig. 6:**
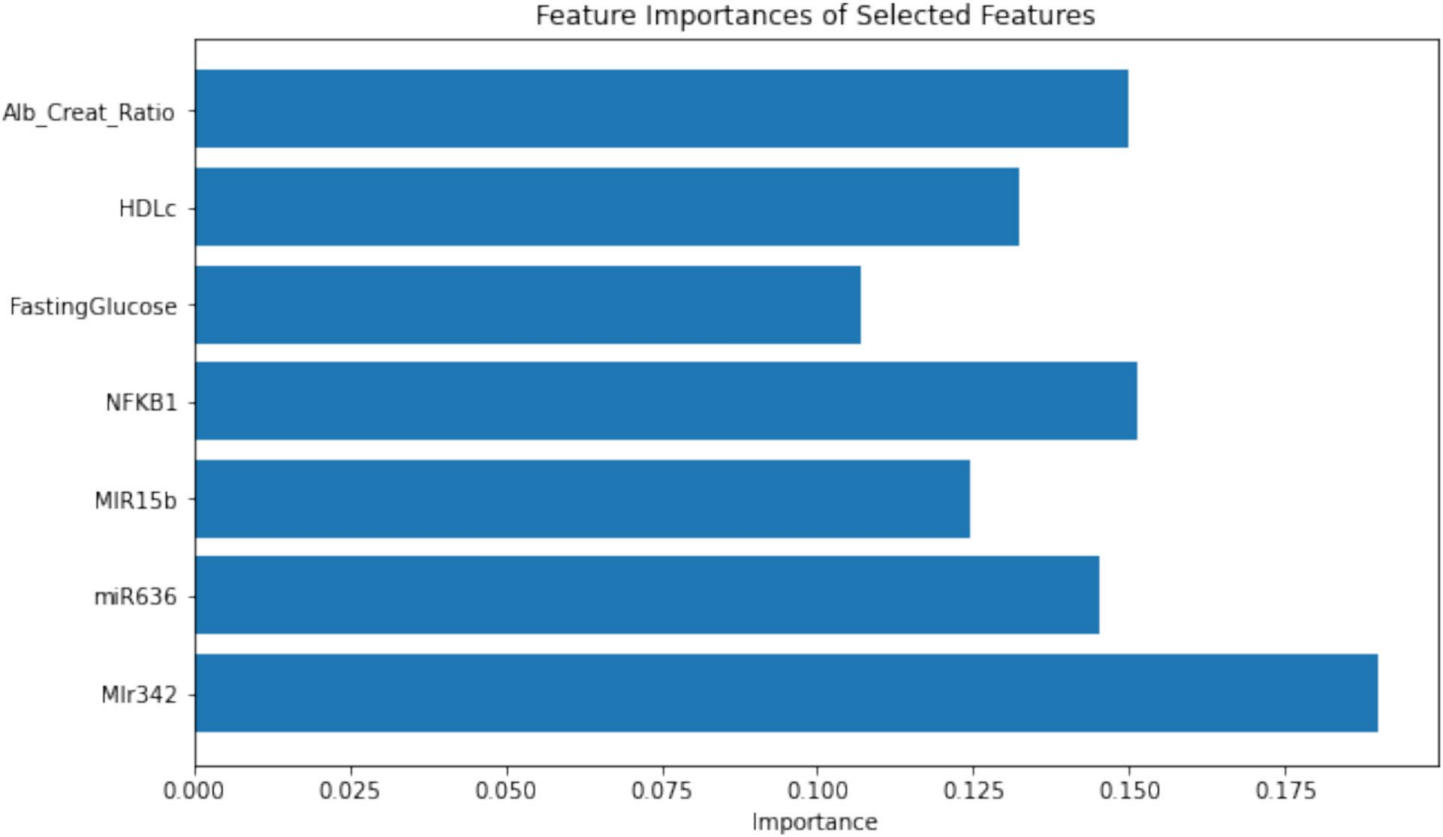
Feature importance for the combined feature group

To evaluate how individual features contribute to the model’s performance, the.

significance of each feature was determined by examining its influence on the model's decision-making. Figure 6 showcases the top important features for the combined model, as identified by the classifier. This analysis is crucial for pinpointing the most impactful features, assisting in informed feature selection, and further model enhancement.

The feature importance for the combined model identified the 5 most contributing features for predicting diabetes stages, comprising three molecular markers—miR342, NFKB1, and miR636—and two biochemical markers, the albumin-to-creatinine ratio and HDLc.

### Model prediction

The initial prediction results with cross-validation on the training set highlighted the top-performing classifiers for each feature group. Table 6 presents the accuracy achieved by each classifier across these groups. Remarkably, the Extra Trees Classifier emerged as the leading performer for all models, followed closely by the RFClassifier, which yielded comparable results.

**Table 6:** Comparison of the performance of the different machine-learning algorithms for each model

Model (Molecular)	Accuracy	AUC	Recall	Precision	F1-Score	MCC
Extra trees classifier	0.9670	0.9984	0.9670	0.9689	0.9667	0.9554
Random Forest	0.9563	0.9945	0.9563	0.9603	0.9549	0.9412
Quadratic discriminant analysis	0.9341	0.9834	0.9341	0.9387	0.9337	0.9117
Naive Bayes	0.9339	0.9849	0.9339	0.9419	0.9342	0.9125
Light gradient boosting machine	0.9177	0.9927	0.9177	0.9248	0.9178	0.8891
Model (Biochemical)						
Extra trees classifier	0.9728	0.9955	0.9728	0.9758	0.9721	0.9636
Random forest	0.9619	0.9952	0.9619	0.9657	0.9605	0.9488
Naive Bayes	0.9617	0.9939	0.9617	0.9659	0.9610	0.9488
Light gradient boosting machine	0.9565	0.9905	0.9565	0.9623	0.9551	0.9422
Quadratic discriminant analysis	0.9071	0.9862	0.9071	0.9225	0.9022	0.8767
Model (Combined)						
Extra Trees Classifier	0.9728	0.9985	0.9728	0.9758	0.9721	0.9636
Random Forest	0.9673	0.9975	0.9673	0.9701	0.9670	0.9558
Quadratic Discriminant Analysis	0.9673	0.9872	0.9673	0.9708	0.9672	0.9564
Naive Bayes	0.9616	0.9872	0.9616	0.9678	0.9621	0.9495
Light Gradient Boosting Machine	0.9562	0.9912	0.9562	0.9597	0.9557	0.9411

Subsequently, the chosen classifiers were applied on the testing set to assess their predictive performance on unseen data. This approach allowed us to apply only the most effective classifiers for each feature group, thereby improving the robustness and reliability of our predictive models. Table 7 provides a summary of the evaluation metrics for the testing set. Per class metrics for the combined model have been also reported (Table 8).

**Table 7:** shows the evaluation metric for the best classifiers on the testing set for each feature group

Model (Molecular)	Accuracy	AUC	Recall	Precision	F1-Score	MCC
Extra Trees Classifier	0.9359	0.9956	0.9359	0.9428	0.9359	0.9142
Model (Biochemical)						
Extra Trees Classifier	0.9615	0.9970	0.9615	0.9655	0.9605	0.9486
Model (Combined: Molecular + Biochemical)						
Extra Trees Classifier	0.9744	0.9989	0.9744	0.9744	0.9744	0.9647

**Table 8:** Shows the evaluation metric per class for the Extra trees classifier for the combined model

Model (Combined)	Precision	Recall	F1-Score
Healthy (class 0)	1.00	1.00	1.00
Prediabetic(class 1)	1.00	1.00	1.00
T2DM without Complications (class 3)	0.96	0.96	0.96
T2DM with Complciations (class 4)	0.93	0.93	0.93
Macro average	0.97	0.97	0.97
Weighted average	0.97	0.97	0.97

In the evaluation results, the Extra Trees classifier demonstrated impressive performance across all models. The molecular model achieved an accuracy of 93.59% with an AUC of 0.9956 (95% CI: [0.988–1.000]), recall of 93.59%, and precision of 94.28%. The biochemical model reached an accuracy of 96.15% and an AUC of 0.9970 (95% CI [0.993–1.000]), with recall of 96.15% and precision of 96.55%. Notably, the combined model of molecular and biochemical features exhibited the highest accuracy at 97.44% and an AUC of 0.9989 (95% CI [0.994–1.000]) along with recall and precision both at 97.44%,,indicating the effectiveness of integrating both feature types in enhancing predictive performance. These results underscore the robustness of the Extra Trees classifier in predicting T2DM stages and its potential for clinical application. Also, the recall and precision across all models indicated strong sensitivity and a low rate of false positives. These metrics provide a more comprehensive understanding of the model’s classification behavior and confirm that its performance is not only accurate but also well-balanced across all classes and suggest that the classifier is not biased toward any particular class and performs reliably in differentiating among the four clinical stages.

### Evaluation of ML models in predicting T2DM

**Fig. 7:**
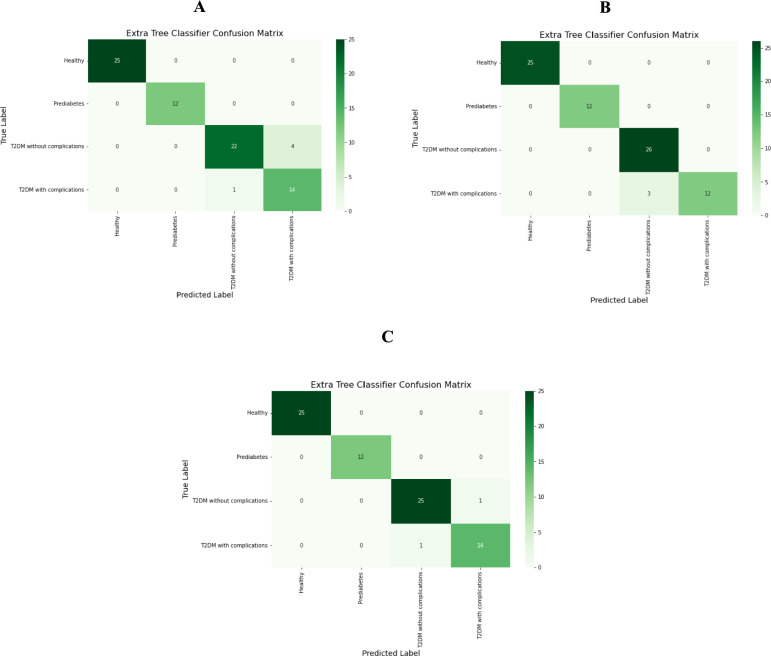
Confusion Matrix for top classifier prediction for each feature group. (**A**) Molecular, (**B**) Biochemical, (**C**) Combined

**Fig. 8:**
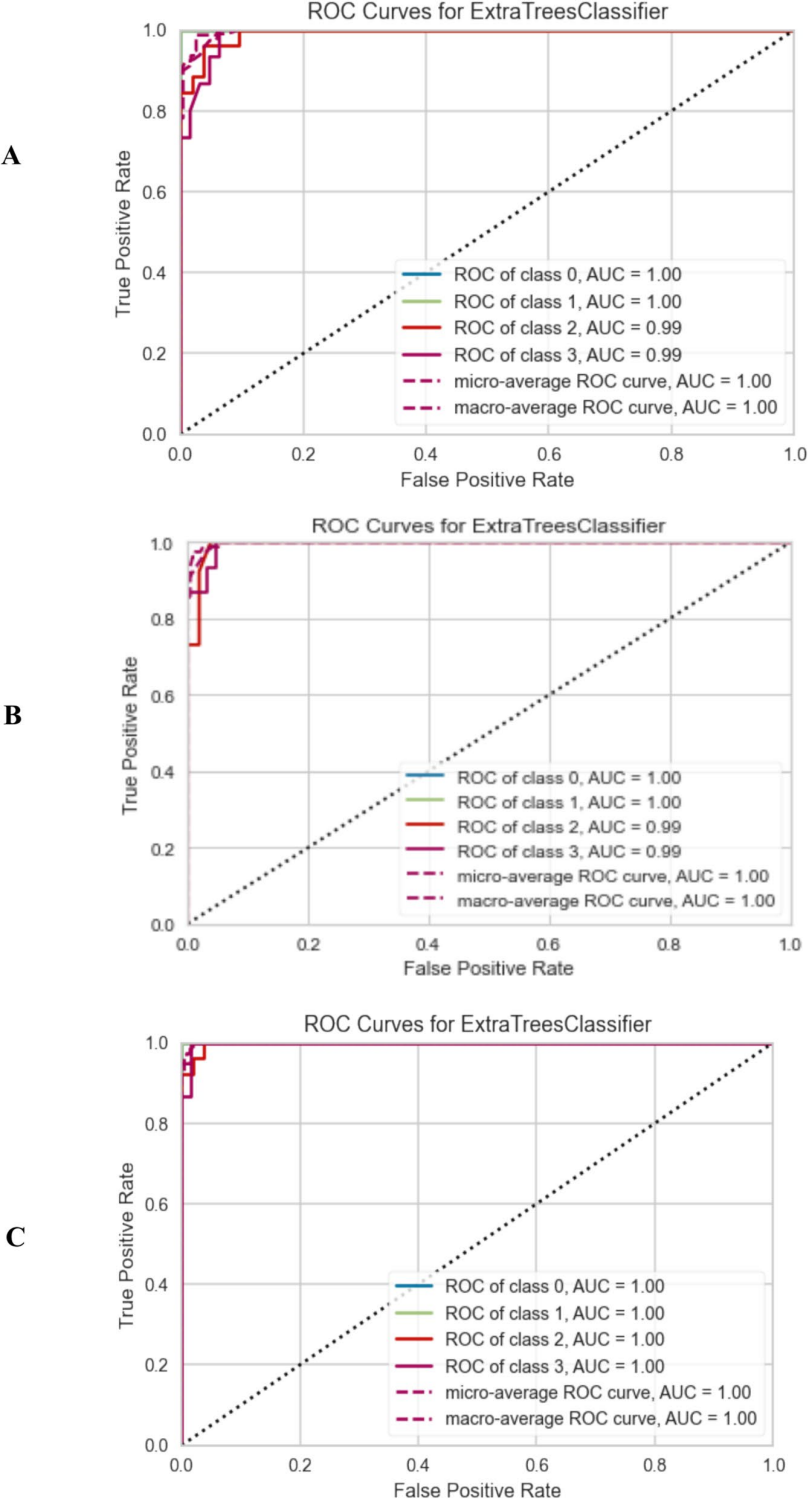
ROC curve for the top-performing classifier for each feature set. (**A**) Molecular, (**B**) Biochemical, (**C**) Combined

The confusion matrix illustrated in Fig. 7 outlines the accuracy of predictions regarding the classification of samples into four categories: healthy, prediabetes, T2DM without complications, and T2DM with complications on the test set for the molecular, biochemical, and combined models. Additionally, the ROC curve Fig. 8 provides insights into the performance of the prediction models, demonstrating their accuracy and ability to differentiate between the various stages of diabetes.

The confusion matrix for the final Extra Trees Classifier for the combined model demonstrated excellent performance across all four health states. All healthy (n = 25) and prediabetes (n = 12) samples in the test set were correctly classified, indicating strong class separability for these groups. For the T2DM without complications class, 25 out of 26 samples were correctly predicted, with only one misclassified as T2DM with complications. Likewise, in the T2DM with complications group, 14 out of 15 samples were correctly classified, with one misclassified as T2DM without complications. These minor misclassifications occurred only between the two T2DM-related classes, suggesting partial overlap in feature space between patients with and without complications. These minor misclassifications imply that while the model effectively captures distinctions among the stages, there may be some shared characteristics between T2DM categories with and without complications.

## Discussion

T2DM is multifactorial metabolic and endocrine disorder that has adverse influence on health. It represents one of the most common chronic diseases. Thus, it is critical to detect prediabetics and diabetics early to avoid diabetic complications [59]. In this research we target to investigate biomarker panel using clinical, bioinformatic, and machine learning for the identification of patients at risk for developing prediabetes or T2DM.

Several studies investigate the pathogenesis of T2DM. It’s well established that insulin resistance is one of the crucial players in transition to prediabetes and diabetes pathogenesis [60, 61].

IGF1R which is a tyrosine kinase, plays a vital role in insulin signaling leading to insulin resistance^.^ [62–64]. Downregulation of IGF1R improved insulin response in diabetic mice [65] and alleviated inflammation of diabetic kidney disease in mice [66] In this study IGF1R was significantly increased when comparing healthy controls and prediabetics, and also when comparing prediabetic with diabetic patients.

mTORC1 enhances insulin resistance, and the production of reactive oxygen species (ROS) causes oxidative damage in membranes, protein, and DNA [67–70]. Also, chronic activation of mTOR inhibits autophagy leading to endoplasmic reticulum stress, mitochondrial dysfunction, inflammation, and β cell failure, which is fundamental in T2DM [71]. In this study, mTOR was significantly increased when comparing the control group versus prediabetics and when comparing prediabetics with diabetics with complications.

RB1CC1 is a key autophagy inducer complex protein that is inhibited by mTOR [72]. Also, RB1CC1 increased the insulin secretion and enhanced islet cell viability [15]. RB1CC1 was downregulated in the urine of diabetic kidney diseased patients compared to the control group [31]. In this study RB1CC1was markedly decreased when comparing prediabetics with diabetics and when comparing diabetics with the complicated diabetic group.

NFKB1 is an important player in inflammatory responses. Chronic inflammation is crucial in the development of insulin resistance, and subsequently, T2DM development [73]. Many pancreatic islets of T2DM patients showed elevated levels of interleukin (IL)−1β and NFKB1 also macrophage hyperinfiltration [74, 75]. Translocation of NFKB1 to the nucleus and binding to promoters of many genes facilitates diabetic nephropathy progression [76]. On the other hand, inhibition of NFKB1 by Boswellic extracts could be a potential therapeutic target in T2DM [77].

In this study, NFKB1 was significantly increased when comparing control with prediabetics, prediabetics with diabetics, and diabetics withdiabetics with complications.

Proto-oncogene receptor tyrosine kinase RET, which binds to glial-derived neurotrophic factor GDNF ligand. Binding of RET to GNDF ligand initiates downstream stimulation of PI3K-AKT-mTOR pathways. RET is fundamental in the neuronal system [78]. Targeting RET with a small ligand, protein, or peptide hinders diabetic retinopathy progression [79]. In this study, RET was significantly elevated when comparing control versus prediabetics, prediabetics versus diabetic patients, and diabetics versus complicated diabetics.

One of the Hsp70 family chaperones concerning diabetes is HSPA1B.HSPA1B plays a crucial role in regulating proteostasis by transporting and folding synthesized polypeptides. Moreover, HSPA1B acts as a quality controller in the ER through checking the correct folding of proteins; it also regulates subsequent degradation of proteins [80]. HSPA1A/HSPA1B represents excellentcandidates for therapeutic studies and experiments, as it is suggested by VIVO data that its loss might be protective in respect of albuminuria [81].

DDX58, also known as retinoic acid-inducible gene-I (RIG-I) is one of the RIG-I-like receptor (RLR) family. This family plays a vital role as a pathogen recognition receptor, as it gives the host an antiviral response and puts immune surveillance into action. Diabetic nephropathy showed upregulation of DDX58 when compared to the control group at protein and transcriptomic levels [82]^.^It was also differentially expressed in obese patients with T2DM [83]

Recently, many studies highlight epigenetic regulators in T2DM. miRNAs represent one of these regulators. miRNAs are major players in the pathogenesis of T2DM, starting with pancreatic development, insulin secretion, and insulin resistance [84]. It has also drawn much attention as a therapeutic target and has been studied in the context of future diagnosis and treatment responses [85].

High levels of miR-15b-5p in T2DM patients’ urine were noted to be associated with high albuminuria, in addition to low eGFR. This represents a crucial factor in diabetic nephropathy progression, making it potential target for therapy [86, 87]. While studying livers of hyperglycemic mice, miR-15b was overexpressed. And insulin sensitivity in the livers of mice was enhanced by the inhibition of miR-15b^.^ [88]

miR611 expression was elevated from the healthy control group to the prediabetic group to T2DM [89]. Also, in the rat model, it was significantly increased in the healthy group compared to the T2DM group [90].

In T2DM patients, initial blood glucose was positively correlated with miR-342–3p [91]. Also in gestational DM, IR and liver gluconeogenesis were inhibited by downregulation of miR-342-3p, which potentiates miR-342 as a therapeutic target in GDM [92]. However, in T regulatory cells, T1DM patients’ miR 342 was downregulated [93].

In the current study there was a significant increase in miR-15b-5p, miR611, and miR-342–5p as we go from the healthy control group to prediabetic to non-complicated T2DM, reaching the maximum level in complicated T2DM patients.

High expression levels of miR-636 were noted during diabetes progression in rats’ renal tissues, in correlation with HbAc1 and the albumin creatinine ratio in urine [94]. Caffeic acid that stimulates autophagy is used for mR-636 inhibition, which enhances glomerular functions in diabetic model rats [95]. In this study, there was a significant increase in miR-636 RQ when we went from prediabetics to non-complicated T2DM, reaching the highest level in complicated T2DM patients.

Machine learning has revolutionized the healthcare sector, emerging as a critical tool for early diagnosis and prediction of the disease. It improves the decision-making process for medical professionals by facilitating rapid and accurate diagnoses of diseases [96].

In this study, we integrated bioinformatics and machine learning techniques to develop a robust predictive model that distinguishes individuals across four health states: healthy, prediabetes, T2DM without complications, and T2DM with complications. By incorporating molecular and biochemical markers, we identify key biomarkers that can discriminate between these disease stages and offer clue aboutthe progression of Type 2 Diabetes Mellitus (T2DM).

The target variable representing the four stages of diabetes was encoded to facilitate effective multi-class classification within the machine learning framework. Specifically, each category was assigned a numerical label: healthy (0), prediabetes (1), T2DM without complications (2), and T2DM with complications (3). This labeling approach ensured that no ordinal relationship was imposed between the classes, treating each health state as a distinct, categorical class. As a result, the machine learning models were able to differentiate between these discrete health conditions, enabling accurate multi-class predictions.

We employed a multi-classifier approach, utilizing Extra Trees Classifier, RFClassifier, Quadratic Discriminant Analysis, Naive Bayes, and Light Gradient Boosting Machine. Our analysis included three models focusing on individual feature types—molecular and biochemical—as well as a combined model. We divided dataset into 70% for training and 30% for testing. We applied Recursive Feature Elimination with Cross-Validation (RFECV) and feature importance analysis for feature selection, which identified five key features contributing significantly to predicting diabetes stages: three molecular markers—miR342, NFKB1, and miR636—and two biochemical markers, namely the albumin-to-creatinine ratio and HDLc. All models were evaluated using fivefold cross-validation to ensure their robustness.

The predictive performance of the molecular, biochemical, and combined models, all utilizing the Extra Trees Classifier, highlights its selection as the top-performing model. This classifier demonstrates a strong capability to accurately and reliably classify health states within the testing set. Each model was assessed using critical metrics, including accuracy, AUC, recall, precision, F1-score, and the MCC, which collectively reflect its effectiveness in classification. While we applied the SMOTE technique to balance the sample sizes across the classes, it remains recommended to use MCC when comparing the performance of different machine learning models.

The molecular model achieved an accuracy of 93.59%, with an impressive AUC of 0.9956, indicating excellent discrimination ability. The model's recall and precision were similarly high, at 93.59% and 94.28%, respectively, resulting in an F1-score of 93.59% and an MCC of 0.9142. These results suggest that the molecular model is robust at capturing relevant biological variations across the four health states.

The biochemical model slightly outperformed the molecular model with an accuracy of 96.15% and an AUC of 0.9970. Its recall, precision, and F1-score were all above 96%, and its MCC was notably high at 0.9486, indicating a strong correlation between predicted and true classes.

Finally, the combined model, which used both molecular and biochemical features, performed the best with an accuracy of 97.44% and an AUC of 0.9989, indicating near-perfect discrimination ability. Its recall, precision, and F1-score were all 97.44%, and MCC was 0.9647, reflecting the highest agreement between predicted and actual health states. This result indicates that combining molecular and biochemical features enhances the model's overall performance, likely due to the complementary information provided by these two feature types. The combined model’s high accuracy and strong MCC suggest that it is well-suited for clinical applications, as it can accurately stratify patients into distinct T2DM progression stages, supporting more tailored and effective patient management strategies.

The confusion matrix for the combined model using the Extra Trees Classifier reveals strong predictive performance across the four health states: healthy, prediabetes, T2DM without complications, and T2DM with complications. All 25 healthy samples and all 12 prediabetes samples were accurately classified, indicating the model’s robustness in identifying these groups. For the T2DM without complications class, 25 out of 26 samples were correctly classified. Only one sample was misclassified as T2DM with complications, suggesting a slight overlap in feature patterns between these two T2DM groups. Similarly, in the T2DM with complications group, 14 samples were correctly classified, with one misclassified as T2DM without complications. These minor misclassifications imply that while the model effectively captures distinctions among the stages, there may be some shared characteristics between T2DM categories with and without complications. Overall, the confusion matrix highlights the model's high accuracy and reliability in predicting each health state, with minimal misclassifications. These results suggest that the combined model effectively captures the key patterns and distinctions among the four health states, supporting its potential utility in clinical or diagnostic applications for stratifying patients based on T2DM progression.

Batch effects were addressed starting from GEO dataset harmonization, GEO2R normalization, statistical adjustment, and final validation of the chosen molecular parameter in the validation cohort (n = 260) (details in supplementary table S5).

Many studies have investigated the use of machine learning methods for predicting diabetes. Kaur and Kumari [84] analyzed risk factors for diabetes using the Pima Indian Diabetes dataset, focusing on female patients, with data provided by the National Institute of Diabetes and Digestive and Kidney Diseases. The dataset, comprising 768 samples with binary classification and eight risk factors, underwent preprocessing to handle outliers and impute missing values using k-nearest neighbor imputation. Feature selection via the Boruta Wrapper algorithm identified four significant attributes. Five classification models were implemented in R, including K-Nearest Neighbor, Neural Network, Support Vector Machine (SVM) with linear and radial basis function kernels, and Multifactor Dimensionality Reduction. Among these, SVM with a linear kernel and K-Nearest Neighbor achieved the highest accuracy scores of 0.90 and 0.92, respectively, highlighting them as effective methods for predicting diabetes risk [97].

Kishor and Chakraborty [98] introduced an advanced healthcare model using machine learning to improve the accuracy and promptness of diabetes diagnosis. This model incorporates five classifiers: logistic regression, K-nearest neighbor, naive Bayes, random Forest, and support vector machine. To refine model performance, they applied the Fast Correlation-Based Filter (FCBF) to exclude irrelevant features and used Synthetic Minority Over-sampling (SMOTE) to address data imbalance. The model's evaluation involved four metrics—accuracy, sensitivity, specificity, and AUC. Results revealed that only a few critical features were needed to enhance model accuracy, with the Random Forest classifier achieving top scores in accuracy (97.81%), sensitivity (99.32%), specificity (98.86%), and AUC (99.35%) [98].

Chen and Pan [99] conducted a study to identify the most effective machine learning model for diabetes prediction, utilizing a dataset with 520 samples and 17 health-related features. They compared eight classification methods, including Support Vector Classifier, Gaussian Naive Bayes, Random Forest, DT, Logistic Regression, Extra Trees Classifier, K-Nearest Neighbors, and XGBoost. Among these, the Extra Trees Classifier demonstrated the highest accuracy at 98.55%, highlighting it as the most accurate and efficient classifier for diabetes diagnosis based on their selected variables [99].

Zou et al. [100] utilized machine learning to predict diabetes in a study based on data.

from hospital examinations in Luzhou, China. They employed J48DT, RF, and Artificial Neural Network (ANN) models, selecting the top-performing methods for further validation to ensure broad applicability in clinical settings. This selection helped in refining techniques for diabetes prediction in diverse population samples [100].

Modak and Jha [101] developed a diabetes prediction model that uses machine learning to aid early diagnosis, potentially reducing complications such as kidney and heart disease. The model leverages a range of algorithms—logistic regression, SVM, Naïve Bayes, and random forest—alongside advanced ensemble methods like XGBoost, LightGBM, CatBoost, AdaBoost, and bagging to improve prediction accuracy and reliability. Using a dataset from Kaggle and implemented in Python, their model was evaluated based on confusion matrix, sensitivity, and accuracy metrics. Among the methods tested, CatBoost was the best performer, achieving a 95.4% accuracy and a 0.99 AUC-ROC score, surpassing XGBoost, which reached 94.3% accuracy and a 0.98 AUC-ROC. This study underscores the potential of ensemble methods in enhancing diabetes prediction performance through robust and precise diagnostics [101].

Abnoosian et al. [102] developed a pipeline-based multi-classification framework to.

predict diabetes status across three categories: diabetic, non-diabetic, and prediabetic, using an imbalanced dataset of Iraqi patients. Their approach involved several pre-processing steps, including duplicate removal, data normalization, feature selection, and missing value imputation, along with k-fold cross-validation. They evaluated various machine learning models, such as k-Nearest Neighbors (k-NN), Support Vector Machine (SVM), DT, RF, AdaBoost, and Gaussian Naïve Bayes (GNB). To address data imbalance, they introduced a weighted ensemble model optimized by AUC. Model performance was further refined using grid search and Bayesian optimization for hyperparameter tuning. Their ensemble model achieved an impressive accuracy of 98.87% and AUC of 0.999, outperforming other classifiers tested in the study [102].

Our biomarker selection aligns with and extends findings from prior large-scale studies while addressing their limitations (supplementary table S6). The biomarker panel (miR342, NFKB1, miR636, albumin-to-creatinine ratio, HDLc) bridges mechanistic depth (autophagy-inflammation axis) and clinical scalability, aligns with established pathways in T2DM pathogenesis while introducing novel insights into disease progression and complications outperforms isolated GWAS/proteomic markers in personalized risk stratification. Even with minor misclassifications, the model provides actionable thresholds for early intervention, as evidenced by validation cohort outcomes. Of note, GWAS has some limitations, as: a) miRNAs are rarely prioritized in GWAS due to their regulatory roles, but transcriptomic studies link miR-342-5p to insulin signaling (e.g., suppression of IRS1 in adipose tissue) and miR-636 to autophagy in diabetic nephropathy. b) While HDLc is a known cardiovascular risk factor, GWAS highlights genetic variants (e.g., CETP) influencing HDL levels rather than HDLc itself as a causal biomarker. Our model leverages HDLc’s dynamic decline with disease progression, consistent with longitudinal studies. Lastly, larger omics cohorts prioritize other variants like TCF7L2 or SLC30A8, which were not addressed here. Future work could harmonize our biomarkers with GWAS loci for polygenic risk scoring.

## Conclusion

In conclusion, our results indicate that integrating machine learning, bioinformatics, and clinical data with biochemical and molecular features shows significant potential to enhance diagnostic precision and staging of diabetes. The classifiers developed in this study effectively differentiated diabetes stages in our cohort, highlighting the promise of multimodal approaches for precision medicine applications. However, these results represent a proof-of-concept requiring rigorous external validation before clinical implementation can be considered. Future work must address key limitations through: (1) Validation on larger, multi-center datasets with diverse demographics, (2) Real-world performance testing in clinical workflows, and (3) Assessment of long-term impact on patient outcomes. Until such validation is completed, this framework should be considered a research tool rather than a clinical solution."

## Supplementary Information

Additional file 1.

Additional file 2.

## Data Availability

No datasets were generated or analysed during the current study.

## References

[1] Abdallah SM, Ayoub AI, Makhlouf MM, Ashour A. Diabetes knowledge, health literacy and diabetes self-care among older adults living with diabetes in Alexandria Egypt. BMC Pub Health. 2024;24(1):2848. 39415165 10.1186/s12889-024-20238-w PMC11481765

[2] Butt MD, Ong SC, Rafiq A, Kalam MN, Sajjad A, Abdullah M, Malik T, Yaseen F, Babar ZU. A systematic review of the economic burden of diabetes mellitus: contrasting perspectives from high and low middle-income countries. J Pharm Policy Pract. 2024;17(1):2322107. 38650677 10.1080/20523211.2024.2322107 PMC11034455

[3] Soliman AR, Hegazy M, Ahmed RM, Abdelghaffar S, Gomaa M, Alwakil S, Soliman D, Sedky L, Shaltout I. Dietary recommendations for people with diabetes in special situations: a position statement report by Arabic association for the study of diabetes and metabolism (AASD). J Health Popul Nutr. 2024;43(1):139. 39227957 10.1186/s41043-024-00619-y PMC11373442

[4] Federation ID. IDF diabetes atlas, tenth. International Diabetes. 2021.

[5] Fowler MJ. Microvascular and macrovascular complications of diabetes. Clinical diabetes. 2008;26(2):77–82.

[6] Tomic D, Shaw JE, Magliano DJ. The burden and risks of emerging complications of diabetes mellitus. Nat Rev Endocrinol. 2022;18(9):525–39. 35668219 10.1038/s41574-022-00690-7 PMC9169030

[7] Bielska A, Niemira M, Kretowski A. Recent highlights of research on miRNAs as early potential biomarkers for cardiovascular complications of type 2 diabetes mellitus. Int J Mol Sci. 2021;22(6):3153. 33808800 10.3390/ijms22063153 PMC8003798

[8] Ramasubbu K, Devi RV. Impairment of insulin signaling pathway PI3K/Akt/mTOR and insulin resistance induced AGEs on diabetes mellitus and neurodegenerative diseases: a perspective review. Mol Cell Biochem. 2023;478(6):1307–24. 36308670 10.1007/s11010-022-04587-x

[9] Yang K, Cao F, Wang W, Tian Z, Yang L. The relationship between HMGB1 and autophagy in the pathogenesis of diabetes and its complications. Front Endocrinol. 2023;29(14):1141516. 10.3389/fendo.2023.1141516 PMC10090453 37065747

[10] Hussein NM, Shehabeldin N, Mohammed AZ, Mohamed HK. Role of LncRNA H19 in the regulation of IGF-1R expression: a possible association between type 2 diabetes and hepatocellular carcinoma: a review article. Med J Cairo Univ. 2022;90(9):1505–13.

[11] Bhardwaj G, Penniman CM, Jena J, Beltran PA, Foster C, Poro K, Junck TL, Hinton AO, Souvenir R, Fuqua JD, Morales PE. Insulin and IGF-1 receptors regulate complex I–dependent mitochondrial bioenergetics and supercomplexes via FoxOs in muscle. J Clin Investig. 2021. 10.1172/JCI146415. 34343133 10.1172/JCI146415 PMC8439595

[12] Iwasaki K, Lalani B, Kahng J, Carapeto P, Sanjines S, Hela F, Abarca C, Tsuji T, Darcy J, Bartke A, Tseng YH. Decreased IGF1R attenuates senescence and improves function in pancreatic β-cells. Front Endocrinol. 2023;27(14):1203534. 10.3389/fendo.2023.1203534 PMC10335398 37441495

[13] Geffken SJ, Moon S, Smith CO, Tang S, Lee HH, Lewis K, Wong CW, Huang Y, Huang Q, Zhao YT, Cai W. Insulin and IGF-1 elicit robust transcriptional regulation to modulate autophagy in astrocytes. Mol Metab. 2022;1(66): 101647. 10.1016/j.molmet.2022.101647 PMC9731889 36503893

[14] Gastol J, Polus A, Biela M, Razny U, Pawlinski L, Solnica B, Kiec-Wilk B. Specific gene expression in type 1 diabetic patients with and without cardiac autonomic neuropathy. Sci Rep. 2020;10(1):5554. 32221364 10.1038/s41598-020-62498-7 PMC7101413

[15] Cui K, Li Z. Identification and analysis of type 2 diabetes-mellitus-associated autophagy-related genes. Front Endocrinol. 2023;8(14):1164112. 10.3389/fendo.2023.1164112 PMC10200926 37223013

[16] Abbaszadeh-Goudarzi K, Radbakhsh S, Pourhanifeh MH, Khanbabaei H, Davoodvandi A, Fathizadeh H, Sahebkar A, Shahrzad MK, Mirzaei H. Circular RNA and diabetes: epigenetic regulator with diagnostic role. Curr Mol Med. 2020;20(7):516–26. 31995005 10.2174/1566524020666200129142106

[17] Yang WonMo YW, Jeong HyoJin JH, Park SeWhan PS, Lee Wan LW. Obesity-induced miR-15b is linked causally to the development of insulin resistance through the repression of the insulin receptor in hepatocytes. 10.1002/mnfr.201500107 26179126

[18] Ye C, Niu J, Zhao Z, Li M, Xu Y, Lu J, Chen Y, Wang W, Ning G, Bi Y, Xu M. Genetic susceptibility, family history of diabetes and healthy lifestyle factors in relation to diabetes: a gene–environment interaction analysis in Chinese adults. J Diabet Investig. 2021;12(11):2089–98. 10.1111/jdi.13577 PMC8565412 33998159

[19] Chaki J, Ganesh ST, Cidham SK, Theertan SA. Machine learning and artificial intelligence based diabetes mellitus detection and self-management: a systematic review. J King Saud Univ-Comput Inf Sci. 2022;34(6):3204–25.

[20] Nuthakki P, Kumar TP. Machine learning-based early detection of diabetes risk factors for improved health management. Multimed Tools Appl. 2024;83:89665–80. 10.1007/s11042-024-18728-5.

[21] Dholariya S, et al. Unveiling the utility of artificial intelligence for prediction, diagnosis, and progression of diabetic kidney disease: an evidence-based systematic review and meta-analysis. Curr Med Res Opin. 2024. 10.1080/03007995.2024.2423737. 39474800 10.1080/03007995.2024.2423737

[22] Flowers E, Stroebel B, Gong X, Lewis KA, Aouizerat BE, Gadgil M, Kanaya AM, Zhang L. Longitudinal associations between microRNAs and weight in the diabetes prevention program. Front Endocrinol. 2024;15:1419812. 10.3389/fendo.2024.1419812. 10.3389/fendo.2024.1419812 PMC11445047 39359416

[23] Stevens PE, Ahmed SB, Carrero JJ, Foster B, Francis A, Hall RK, Herrington WG, Hill G, Inker LA, Kazancıoğlu R, Lamb E. KDIGO 2024 clinical practice guideline for the evaluation and management of chronic kidney disease. Kidney Int. 2024;105(4):S117-314. 38490803 10.1016/j.kint.2023.10.018

[24] Levin A, Ahmed SB, Carrero JJ, Foster B, Francis A, Hall RK, Herrington WG, Hill G, Inker LA, Kazancıoğlu R, Lamb E. Executive summary of the KDIGO 2024 clinical practice guideline for the evaluation and management of chronic kidney disease: known knowns and known unknowns. Kidney Int. 2024;105(4):684–701. 38519239 10.1016/j.kint.2023.10.016

[25] Wood M, Bando H, Ebe K. Standards of Care in Diabetes 2025: Diabetes-Associated Autoantibodies.

[26] Talukder MA, Islam MM, Uddin MA, Kazi M, Khalid M, Akhter A, Ali MM. Toward reliable diabetes prediction: Innovations in data engineering and machine learning applications. Digit Health. 2024;10:20552076241271868. 39175924 10.1177/20552076241271867 PMC11339751

[27] Dharmarathne G, Jayasinghe TN, Bogahawaththa M, Meddage DP, Rathnayake U. A novel machine learning approach for diagnosing diabetes with a self-explainable interface. Healthc Anal. 2024;1(5): 100301.

[28] Montaser E, Farhy LS, Rich SS. Enhancing type 1 diabetes immunological risk prediction with continuous glucose monitoring and genetic profiling. Diabet Technol Ther. 2025;27(4):292–300. 10.1089/dia.2024.0496. 10.1089/dia.2024.0496 PMC13354271 39686752

[29] Montaser E, Shah VN. Prediction of incident diabetic retinopathy in adults with type 1 diabetes using machine learning approach: an exploratory study. J Diabet Sci Technol. 2024;28:19322968241292370. 10.1177/19322968241292369 PMC11571610 39465559

[30] Ali HS, Boshra MS, Agwa SH, Hakeem MS, Meteini MS, Matboli M. Identification of a multi-messenger RNA signature as type 2 diabetes mellitus candidate genes involved in crosstalk between inflammation and insulin resistance. Biomolecules. 2022;12(9):1230. 36139069 10.3390/biom12091230 PMC9496026

[31] Matboli M, Azazy AE, Adel S, Bekhet MM, Eissa S. Evaluation of urinary autophagy transcripts expression in diabetic kidney disease. J Diabet Complic. 2017;31(10):1491–8. 10.1016/j.jdiacomp.2017.06.009 28760651

[32] Yasuda-Yamahara M, Kume S, Maegawa H. Roles of mTOR in diabetic kidney disease. Antioxidants. 2021;10:321. 33671526 10.3390/antiox10020321 PMC7926630

[33] Matboli M, Kamel MM, Essawy N, Bekhit MM, Abdulrahman B, Mohamed GF, Eissa S. Identification of novel insulin resistance related ceRNA network in T2DM and its potential editing by CRISPR/Cas9. Int J Mol Sci. 2021;22(15):8129. 34360895 10.3390/ijms22158129 PMC8348752

[34] Madeira F, Madhusoodanan N, Lee J, Eusebi A, Niewielska A, Tivey AR, Meacham S, Lopez R, Butcher S. Using EMBL-EBI services via web interface and programmatically via web services. Curr Protoc. 2024;4(6): e1065. 38857087 10.1002/cpz1.1065

[35] Diagnosis and classification of diabetes: standards of care in diabetes—2024. Diabetes Care 47, no. Supplement_1 (2024): S20-S42 10.2337/dc24-S002 PMC10725812 38078589

[36] Wallace TM, Levy JC, Matthews DR. Use and abuse of HOMA modeling. Diabet Care. 2004;27(6):1487–95. 10.2337/diacare.27.6.1487 15161807

[37] Matthews DR, Hosker JP, Rudenski AS, Naylor BA, Treacher DF, Turner R. Homeostasis model assessment: insulin resistance and β-cell function from fasting plasma glucose and insulin concentrations in man. Diabetologia. 1985;28:412–9. 3899825 10.1007/BF00280883

[38] Royston P. Multiple imputation of missing values. Stand Genom Sci. 2004;4(3):227–41.

[39] Vinutha HP, Poornima B, Sagar BM. Detection of outliers using interquartile range technique from intrusion dataset. InInformation and decision sciences: Proceedings of the 6th international conference on ficta 2018; pp. 511–518. Springer Singapore.

[40] Dash CS, Behera AK, Dehuri S, Ghosh A. An outliers detection and elimination framework in classification task of data mining. Decis Anal J. 2023;1(6): 100164.

[41] Kumar S, Chong I. Correlation analysis to identify the effective data in machine learning: Prediction of depressive disorder and emotion states. Int J Environ Res Public Health. 2018;15(12):2907. 30572595 10.3390/ijerph15122907 PMC6313491

[42] Ali PJ, Faraj RH, Koya E, Ali PJ, Faraj RH. Data normalization and standardization: a technical report. Mach Learn Tech Rep. 2014;1(1):1–6.

[43] Rahman MM, Davis DN. Addressing the class imbalance problem in medical datasets. Int J Mach Learn Comput. 2013;3(2):224.

[44] Chawla NV, Bowyer KW, Hall LO, Kegelmeyer WP. SMOTE: synthetic minority over-sampling technique. J Artif Intell Res. 2002;1(16):321–57.

[45] Kuhn M. Applied predictive modeling.

[46] Anguita D, Ghelardoni L, Ghio A, Oneto L, Ridella S. The’K’in K-fold Cross Validation. InESANN. 2012;102:441–6.

[47] Kovalerchuk B. Enhancement of cross validation using hybrid visual and analytical means with Shannon function. In: Beyond traditional probabilistic data processing techniques: interval, fuzzy etc. Methods and their applications. Cham: Springer; 2020.

[48] Géron A. Hands-on machine learning with Scikit-Learn, Keras, and TensorFlow. O'Reilly Media, Inc. 2022; 4

[49] Breiman L. Random forests. Mach Learn. 2001;45:5–32.

[50] Svetnik V, Liaw A, Tong C, Culberson JC, Sheridan RP, Feuston BP. Random forest: a classification and regression tool for compound classification and QSAR modeling. J Chem Inf Comput Sci. 2003;43(6):1947–58. 14632445 10.1021/ci034160g

[51] Liaw A. Classification and regression by randomForest. R news. 2002.

[52] El-Sofany H, El-Seoud SA, Karam OH, Abd El-Latif YM, Taj-Eddin IA. A proposed technique using machine learning for the prediction of diabetes disease through a mobile app. Int J Intell Syst. 2024;2024(1):6688934.

[53] Ahamed BS. Prediction of type-2 diabetes using the LGBM classifier methods and techniques. Turkish J Comput Math Educ (TURCOMAT). 2021;12(12):223–31.

[54] Zhu T, Li K, Chen J, Herrero P, Georgiou P. Dilated recurrent neural networks for glucose forecasting in type 1 diabetes. J Healthc Inf Res. 2020;4:308–24. 10.1007/s41666-020-00068-2 PMC8982716 35415447

[55] Eker AM, Dikmen M, Cambazoğlu S, Düzgün ŞH, Akgün H. Evaluation and comparison of landslide susceptibility mapping methods: a case study for the Ulus district, Bartın, northern Turkey. Int J Geogr Inf Sci. 2015;29(1):132–58.

[56] Powers DM. Evaluation: from precision, recall and F-measure to ROC, informedness, markedness and correlation. arXiv preprint arXiv:2010.16061. 2020.

[57] McHugh ML. Interrater reliability: the kappa statistic. Biochem Med. 2012;22(3):276–82. PMC3900052 23092060

[58] Sokolova M, Lapalme G. A systematic analysis of performance measures for classification tasks. Inf Process Manage. 2009;45(4):427–37.

[59] Nawaz F, Ramzan M, Mehmood K, Khan HU, Khan SH, Bhutta MR. Early detection of diabetic retinopathy using machine intelligence through deep transfer and representational learning. Comput Mater Continua. 2021. 10.32604/cmc.2020.012887.

[60] Stanciu SM, Jinga M, Miricescu D, Stefani C, Nica RI, Stanescu-Spinu II, Vacaroiu IA, Greabu M, Nica S. mTOR dysregulation, insulin resistance, and hypertension. Biomedicines. 2024;12(8):1802. 39200267 10.3390/biomedicines12081802 PMC11351979

[61] Amin NG, Rahim AA, Rohoma K, Elwafa RA, Dabees HM, Elrahmany S. The relation of mTOR with diabetic complications and insulin resistance in patients with type 2 diabetes mellitus. Diabetol Metab Syndr. 2024;16(1):222. 39261960 10.1186/s13098-024-01450-5 PMC11389252

[62] Lewitt MS, Dent MS, Hall K. The insulin-like growth factor system in obesity, insulin resistance and type 2 diabetes mellitus. J Clin Med. 2014;3(4):1561–74. 26237614 10.3390/jcm3041561 PMC4470198

[63] Yan Y, Hu F, Wu W, Ma R, Huang H. Expression characteristics of proteins of IGF-1R, p-Akt, and survivin in papillary thyroid carcinoma patients with type 2 diabetes mellitus. Medicine. 2017;96(12): e6393. 28328831 10.1097/MD.0000000000006393 PMC5371468

[64] Poulaki V, Joussen AM, Mitsiades N, Mitsiades CS, Iliaki EF, Adamis AP. Insulin-like growth factor-I plays a pathogenetic role in diabetic retinopathy. Am J Pathol. 2004;165(2):457–69. 15277220 10.1016/S0002-9440(10)63311-1 PMC1618554

[65] Engberding N, San Martín A, Martin-Garrido A, Koga M, Pounkova L, Lyons E, Lassègue B, Griendling KK. Insulin-like growth factor-1 receptor expression masks the antiinflammatory and glucose uptake capacity of insulin in vascular smooth muscle cells. Arterioscler Thromb Vasc Biol. 2009;29(3):408–15. 19122171 10.1161/ATVBAHA.108.181727 PMC2713108

[66] Li J, Dong R, Yu J, Yi S, Da J, Yu F, Zha Y. Inhibitor of IGF1 receptor alleviates the inflammation process in the diabetic kidney mouse model without activating SOCS2. Drug Des Dev Ther. 2018;11:2887–96. 10.2147/DDDT.S171638 PMC6141121 30254418

[67] Vergès B, Cariou B. mTOR inhibitors and diabetes. Diabet Res Clin Pract. 2015;110(2):101–8. 10.1016/j.diabres.2015.09.014 26421362

[68] Yin X, Xu Z, Zhang Z, Li L, Pan Q, Zheng F, Li H. Association of PI3K/AKT/mTOR pathway genetic variants with type 2 diabetes mellitus in Chinese. Diabet Res Clin Practice. 2017;128:127–35. 10.1016/j.diabres.2017.04.002 28477532

[69] Cheon SY, Cho K. Lipid metabolism, inflammation, and foam cell formation in health and metabolic disorders: targeting mTORC1. J Mol Med. 2021;99(11):1497–509. 34312684 10.1007/s00109-021-02117-8

[70] Zoncu R, Efeyan A, Sabatini DM. mTOR: from growth signal integration to cancer, diabetes and ageing. Nat Rev Mol Cell Biol. 2011;12(1):21–35. 21157483 10.1038/nrm3025 PMC3390257

[71] Rocha M, Apostolova N, Diaz-Rua R, Muntane J, Victor VM. Mitochondria and T2D: role of autophagy, ER stress, and inflammasome. Trends Endocrinol Metab. 2020;31(10):725–41. 32265079 10.1016/j.tem.2020.03.004

[72] Yao J, Jia L, Khan N, Lin C, Mitter SK, Boulton ME, Dunaief JL, Klionsky DJ, Guan JL, Thompson DA, Zacks DN. Deletion of autophagy inducer RB1CC1 results in degeneration of the retinal pigment epithelium. Autophagy. 2015;11(6):939–53. 26075877 10.1080/15548627.2015.1041699 PMC4502815

[73] Sifuentes-Franco S, Pacheco-Moisés FP, Rodríguez-Carrizalez AD, Miranda-Díaz AG. The role of oxidative stress, mitochondrial function, and autophagy in diabetic polyneuropathy. J Diabet Res. 2017;2017(1):1673081. 10.1155/2017/1673081 PMC5674726 29204450

[74] Margaryan S, Kriegova E, Fillerova R, Smotkova Kraiczova V, Manukyan G. Hypomethylation of IL1RN and NFKB1 genes is linked to the dysbalance in IL1β/IL-1Ra axis in female patients with type 2 diabetes mellitus. PLoS ONE. 2020;15(5): e0233737. 32470060 10.1371/journal.pone.0233737 PMC7259508

[75] Raza W, Guo J, Qadir MI, Bai B, Muhammad SA. qPCR Analysis reveals association of differential expression of SRR, NFKB1, and PDE4B genes with type 2 diabetes mellitus. Front Endocrinol. 2022;3(12): 774696. 10.3389/fendo.2021.774696 PMC8761634 35046895

[76] Guo M, Gao J, Jiang L, Dai Y. Astragalus polysaccharide ameliorates renal inflammatory responses in a diabetic nephropathy by suppressing the TLR4/NF-κB pathway. Drug Des Dev Ther. 2023;31:2107–18. 10.2147/DDDT.S411211 PMC10363349 37489175

[77] Ammon HP. Inhibition of NFĸB-activation as a possible strategy to prevent/treat diabetes mellitus? effects of boswellic extracts and boswellic acids. J Clin Immunol Res Ther. 2023;2(1):1.

[78] Addeo A, Miranda-Morales E, den Hollander P, Friedlaender A, Sintim HO, Wu J, Mani SA, Subbiah V. RET aberrant cancers and RET inhibitor therapies: current state-of-the-art and future perspectives. Pharmacol Ther. 2023;1(242): 108344. 10.1016/j.pharmthera.2023.108344 PMC10141525 36632846

[79] Xu B, Zhang H, Zhu M, Le YZ. Critical role of trophic factors in protecting müller glia implications to neuroprotection in age-related macular degeneration, diabetic retinopathy, and anti-VEGF therapies. In: Retinal degenerative diseases: mechanisms and experimental therapy. Cham: Springer International Publishing; 2019. 10.1007/978-3-030-27378-1_77 31884656

[80] Klyosova E, Azarova I, Buikin S, Polonikov A. Differentially expressed genes regulating glutathione metabolism, protein-folding, and unfolded protein response in pancreatic β-Cells in type 2 diabetes mellitus. Int J Mol Sci. 2023;24(15):12059. 37569434 10.3390/ijms241512059 PMC10418503

[81] Bulkescher R. Effects of dicarbonyl stress in the absence of HSPA1A/HSPA1B in endothelial cells and STZ-induced diabetic mice on the development of diabetic nephropathy (Doctoral dissertation).

[82] Lei L, Bai Y, Fan Y, Li Y, Jiang H, Wang J. Comprehensive diagnostics of diabetic nephropathy by transcriptome RNA sequencing. Diabet Metab Syndr Obes Targ Ther. 2022;1:3069–80. 10.2147/DMSO.S371026 PMC9553241 36237968

[83] An T, Zhang J, Liu YF, Wu YX, Lian J, Wang TY, Hu YY, Zhu JJ, Huang J, Zhao DD, Mo FF. Combined analysis of whole-exon sequencing and lncRNA sequencing in type 2 diabetes mellitus patients with obesity. J Cell Mol Med. 2020;24(4):2451–63. 31957265 10.1111/jcmm.14932 PMC7028848

[84] Kaur P, Kotru S, Singh S, Behera BS, Munshi A. Role of miRNAs in the pathogenesis of T2DM, insulin secretion, insulin resistance, and β cell dysfunction: the story so far. J Physiol Biochem. 2020;76(4):485–502. 32749641 10.1007/s13105-020-00760-2

[85] Mahmoud HS, Esmail OE, Abdel-Raouf W. Role of MicroRNA-224 in the field of diabetes: a comprehensive review. ERU Res J. 2024. 10.21608/erurj.2024.213260.1032.

[86] Tsai YC, Kuo MC, Hung WW, Wu LY, Wu PH, Chang WA, Kuo PL, Hsu YL. High glucose induces mesangial cell apoptosis through miR-15b-5p and promotes diabetic nephropathy by extracellular vesicle delivery. Mol Ther. 2020;28(3):963–74. 31991106 10.1016/j.ymthe.2020.01.014 PMC7054723

[87] Yang Z, Song D, Wang Y, Tang L. lncRNA MALAT1 promotes diabetic nephropathy progression via miR-15b-5p/TLR4 signaling Axis. J Immunol Res. 2022;2022(1):8098001. 35910856 10.1155/2022/8098001 PMC9334040

[88] Li WD, Xia JR, Lian YS. MiR-15b can target insulin receptor to regulate hepatic insulin signaling in mice. Anim Cells Syst. 2019;23(2):82–9. 10.1080/19768354.2019.1583125 PMC6440518 30949394

[89] Ali HS, Kamel MM, Agwa SH, Hakeem MS, Meteini MS, Matboli M. Analysis of mRNA-miRNA-lncRNA differential expression in prediabetes/type 2 diabetes mellitus patients as potential players in insulin resistance. Front Endocrinol. 2023;8(14):1131171. 10.3389/fendo.2023.1131171 PMC10200895 37223012

[90] Matboli M, Al-Amodi HS, Khaled A, Khaled R, Roushdy MM, Ali M, Diab GI, Elnagar MF, Elmansy RA, Tahmed HH, Ahmed EM. Comprehensive machine learning models for predicting therapeutic targets in type 2 diabetes utilizing molecular and biochemical features in rats. Front Endocrinol. 2024;15:1384984. 10.3389/fendo.2024.1384984 PMC11157016 38854687

[91] He L, Bao T, Yang Y, Wang H, Gu C, Chen J, Zhai T, He X, Wu M, Zhao L, Tong X. Exploring the pathogenesis of type 2 diabetes mellitus intestinal damp-heat syndrome and the therapeutic effect of gegen Qinlian decoction from the perspective of exosomal miRNA. J Ethnopharmacol. 2022;1(285): 114786. 10.1016/j.jep.2021.114786 34763043

[92] Sun Y, Yu Z, Zhang Y, Wang H, Chi Z, Chen X, Xu D. Downregulation of microRNA-342–3p eases insulin resistance and liver gluconeogenesis via regulating Rfx3 in gestational diabetes mellitus. Crit RevTM Eukaryot Gene Expr. 2022;32:6. 10.1615/CritRevEukaryotGeneExpr.2022043275 35997120

[93] Hezova R, Slaby O, Faltejskova P, Mikulkova Z, Buresova I, Raja KM, Hodek J, Ovesna J, Michalek J. microRNA-342, microRNA-191 and microRNA-510 are differentially expressed in T regulatory cells of type 1 diabetic patients. Cell Immunol. 2010;260(2):70–4. 19954774 10.1016/j.cellimm.2009.10.012

[94] Wang LP, Gao YZ, Song B, Yu G, Chen H, Zhang ZW, Yan CF, Pan YL, Yu XY. MicroRNAs in the progress of diabetic nephropathy: a systematic review and meta-analysis. Evid-Based Complement Altern Med. 2019;2019(1):3513179. 10.1155/2019/3513179 PMC6431481 30984273

[95] Salem AM, Ragheb AS, Hegazy MG, Matboli M, Eissa S. Caffeic acid modulates miR-636 expression in diabetic nephropathy rats. Indian J Clin Biochem. 2019;1(34):296–303. 10.1007/s12291-018-0743-0 PMC6660537 31391719

[96] Debal DA, Sitote TM. Chronic kidney disease prediction using machine learning techniques. J Big Data. 2022;9(1):1–9.

[97] Kaur H, Kumari V. Predictive modelling and analytics for diabetes using a machine learning approach. Appl Comput Inf. 2022;18(1/2):90–100.

[98] Kishor A, Chakraborty C. Early and accurate prediction of diabetics based on FCBF feature selection and SMOTE. Int J Syst Assur Eng Manag. 2021;23:1–9.

[99] Chen P, Pan C. Diabetes classification model based on boosting algorithms. BMC Bioinform. 2018;19:1–9. 10.1186/s12859-018-2090-9 PMC5872396 29587624

[100] Zou Q, Qu K, Luo Y, Yin D, Ju Y, Tang H. Predicting diabetes mellitus with machine learning techniques. Front Genet. 2018;6(9):515. 10.3389/fgene.2018.00515 PMC6232260 30459809

[101] Modak SK, Jha VK. Diabetes prediction model using machine learning techniques. Multimed Tools Appl. 2024;83(13):38523–49.

[102] Abnoosian K, Farnoosh R, Behzadi MH. Prediction of diabetes disease using an ensemble of machine learning multi-classifier models. BMC Bioinform. 2023;24(1):337. 10.1186/s12859-023-05465-z PMC10496262 37697283

