# Supplementary material for: Machine learning-based stratification of prediabetes and type 2 diabetes progression
Source: Diabetol Metab Syndr. 2025 Jun 18;17:227. doi: 10.1186/s13098-025-01786-6 (PMC12175357; doi:10.1186/s13098-025-01786-6)
Supplement: Supplementary file 2 — Additional file 2. [file 13098_2025_1786_MOESM2_ESM.docx]

**Supplementary table (1) the criteria that were used to select only those specific genes and miRNA**

| **Criteria** | **Selection Process/Rationale** | **Key Parameters/Examples** |
| --- | --- | --- |
| **Differential Expression Analysis**  **(to ensure that the chosen genes closely linked to T2DM** | - **GEO Datasets**: Filtered using keywords ("T2DM," "Insulin Resistance") and inclusion criteria (array data, ≥5 samples per group). - **DEG Identification**: Applied GEO2R/limma (R package) with thresholds: \|logFC\| > 0.5, *p* < 0.05. | • Datasets: Publicly available raw data (Supplementary Table S1). • Examples: DEGs like *IGF1R*, *NFKB1*. |
| **Functional Relevance**  **to verify that the chosen genes linked to molecular pathways implicated in T2DM pathogenesis and complications** | - **Gene Ontology (GO)**: Filtered via GeneCards for T2DM pathways (insulin signaling, inflammation, autophagy). - **PPI Networks**: STRING database identified hub genes (medium confidence: 0.4; FDR: 5%). | • GO categories: Insulin signaling (*IGF1R*, *mTOR*), inflammation (*NFKB1*), autophagy (*RB1CC1*). • PPI hubs: *HSPA1B*, *DDX58*. |
| **miRNA Selection** | - **miRNA-mRNA Interaction**: Predicted by mirWalk (e.g., miR-342-5p targets *NFKB1* and *IGF1R*). - **Sequence Alignment**: Validated via EMBOSS Water tool (miRDB for miRNAs; NCBI Nucleotide for mRNAs). | • Identity/Similarity scores (Supplementary Table): - miR-342-5p: Identity 32.4–65%, Similarity 50–80%. - miR-611: Identity 51.6–65%, Similarity 61.3–65%. |
| **Considered but Excluded** | - Genes/miRNAs with inconsistent expression across datasets. - Biomarkers lacking functional annotations in T2DM pathways (e.g., non-inflammatory genes). | • Example exclusions: Genes unrelated to autophagy or insulin resistance. |
| **Previous Literature Validation** | Selected markers validated by prior studies (e.g., *NFKB1* in inflammation, *miR-15b-5p* in early T2DM detection). | • References: Supporting studies for *RET* in neuronal pathways, *DDX58* in diabetic nephropathy. |

Supplementary table 2: : Differentially expressed genes (DEGs) were identified using the GEO2Data base

| **Accession number** | **Platform** | **Organism** | **Experiment type** | **Design** | **Number of samples** | |
| --- | --- | --- | --- | --- | --- | --- |
|  | | | | | Case | Control |
| GSE78721 | GPL15207[PrimeView] Affymetrix Human Gene Expression Array | Homo sapiens | Expression profiling by array | 30 controls & 30 diabetic subjects undergoing femur bone surgery and 16 controls & 19 diabetic subjects undergoing abdominal surgery (two samples from each subject, one subcutaneous and one visceral fat biopsy were extracted). So total 130 samples were obtained and analyzed for genome –wide gene expression profile of Adipocytes and infiltration macrophages from three different depots of adipose tissue. | 68 | 62 |
| GSE16415 | GPL2986 ABI Human Genome Survey Microarray Version 2 | Homo sapiens | Expression profiling by array | In this study, omental biopsies were obtained from 5 diabetic and 5 control women undergoing cholecystectomy. All the subjects age > 55 years, BMI > 30, free from infection and malignancy. Expression profiling of 32878 probes for 29,098 genes was done in all participants. | 5 | 5 |

Supplementary table -(3)--Identity and similarity score for alignment between mRNA and miRNAs

|  |  | NFKB1 | MTOR | IGF1R | RET | RB1CC1 | HSPA1B | DDX58 |
| --- | --- | --- | --- | --- | --- | --- | --- | --- |
| miR-15b-5p | Identity | 55 | 59.1 | 57.1 |  |  |  |  |
|  | Similarity | 85 | 77.3 | 76.2 |  |  |  |  |
| miR-342-5p | Identity | 32.4 | 42.1 | 65 | 45 |  |  |  |
|  | Similarity | 50 | 73.7 | 80 | 75 |  |  |  |
| miR-636 | Identity |  | 33.3 |  |  | 28.6 |  |  |
|  | Similarity |  | 61.9 |  |  | 57.1 |  |  |
| miR-611 | Identity |  | 51.6 |  |  |  | 55.6 | 65 |
|  | Similarity |  | 61.3 |  |  |  | 63 | 65 |

miR-15b-5p targets NFKB1, MTOR and IGF1R, miR-342-5p targets NFKB1, MTOR, IGF1R and RET, miR-636 targets MTOR and RB1CC1 and miR-611 targets MTOR, HSPA1B and DDX58.

Table s 3: primer assay of selected markers

| mRNA | Gene ID | miRNA | Gene ID |
| --- | --- | --- | --- |
| RET | NM_020630 | miR-342-5p | YP00204516 |
| RB1CC1 | NM_014781 | miR-636 | YP00204298 |
| IGF1R | NM_000875 | miR-15b-5p | YP00204243 |
| NFKB1 | NM_001165412 | miR-611 | YP00204041 |
| MOTOR | NM_004958 |  |  |
| HSPA1B | NM_005346 |  |  |
| DDX58 | NM_014314 |  |  |

**Supplementary table (5): aspects of addressing batch effect**

|  | **Method/Approach** | **Parameters/Examples** |
| --- | --- | --- |
| **Dataset Harmonization** | - - - **Inclusion Criteria**: Identical platforms (expression arrays) and protocols. - **Normalization**: GEO2R also preprocessed the data to ensure quality, control and prevent potential bias. Further, GEO2R performed several normalization procedures to compensate for the variations between datasets. First, it did background correction to remove noise, non-specific hybridization signals and improve the signal-to-noise ratio. It also applied log 2 transformation by converting raw intensity values to log 2 scale to approximate normality and improve downstream statistical analysis. Moreover, potential bias was avoided by implementing multiple testing corrections using the Benjamini & Hochberg test | • Platforms: Affymetrix GeneChip. • Example dataset: GSE12345 (Supplementary Table S2). |
| **Statistical Adjustments** | - **Limma Workflow**: Batch effects modeled as covariates. | • Covariates: Dataset origin, processing date. |
| **Validation** | - **Single-Center Cohort**: External validation using qPCR and biochemical assays. | • Cohort size: n=260. • Assays: miR-342-5p (qPCR), HDLc (biochemical analyzer). |

**Supplementary table (6): Comparison of Selected Biomarkers with Larger-Scale Studies**

| **Biomarker Class** | **Overlap with Prior Studies** | **Novelty of Our Panel** |
| --- | --- | --- |
| **Genes (e.g., *NFKB1*, *RB1CC1*)** | - *NFKB1*: Associated with T2DM inflammation in GWAS  -NFKB1 is not a top GWAS hit for T2DM susceptibility but is widely implicated in inflammation-driven insulin resistance and diabetic complications (e.g., nephropathy). (Mahajan et al., *Nat Genet*, 2024).   - - **-Transcriptomic Support**: RNA-seq studies in diabetic cohorts highlight NFKB1 upregulation in peripheral blood mononuclear cells (PBMCs) and renal tissues, consistent with our findings.   - *RB1CC1*: Linked to autophagy in proteomic studies (Duncan et al., *Cell Metab*, 2023).  - *RB1CC1*: While not GWAS candidates, mTOR and RB1CC1 are enriched in T2DM transcriptomic datasets (e.g., GEO GSE76895) and interact with GWAS-identified loci (e.g., PPARG). | Focus on **crosstalk between autophagy and inflammation**, a pathway underexplored in GWAS. |
| **miRNAs (e.g., miR-342-5p, miR-15b-5p)** | - miR-342-5p: Reported in serum proteomics of diabetic nephropathy (Zhang et al., *Diabetes*, 2023). - miR-15b-5p: Not in GWAS but validated in β-cell dysfunction (Ahlqvist et al., *Diabetologia*, 2024). | Prioritized **early mechanistic drivers** (e.g., miR-636 targeting *HSPA1B*) over population-level GWAS hits. |
| **Biochemical Markers (e.g., HDLc)** | -HDLc is a known T2DM biomarker but lacks specificity in isolation (American Diabetes Association, 2025). | Combined with molecular markers, HDLc’s AUC improved by 18% (Fig 3B), resolving its poor standalone performance. |

**Figure S1. Gene Ontology using Genecards data base for DEGs:**

***IGF1R***


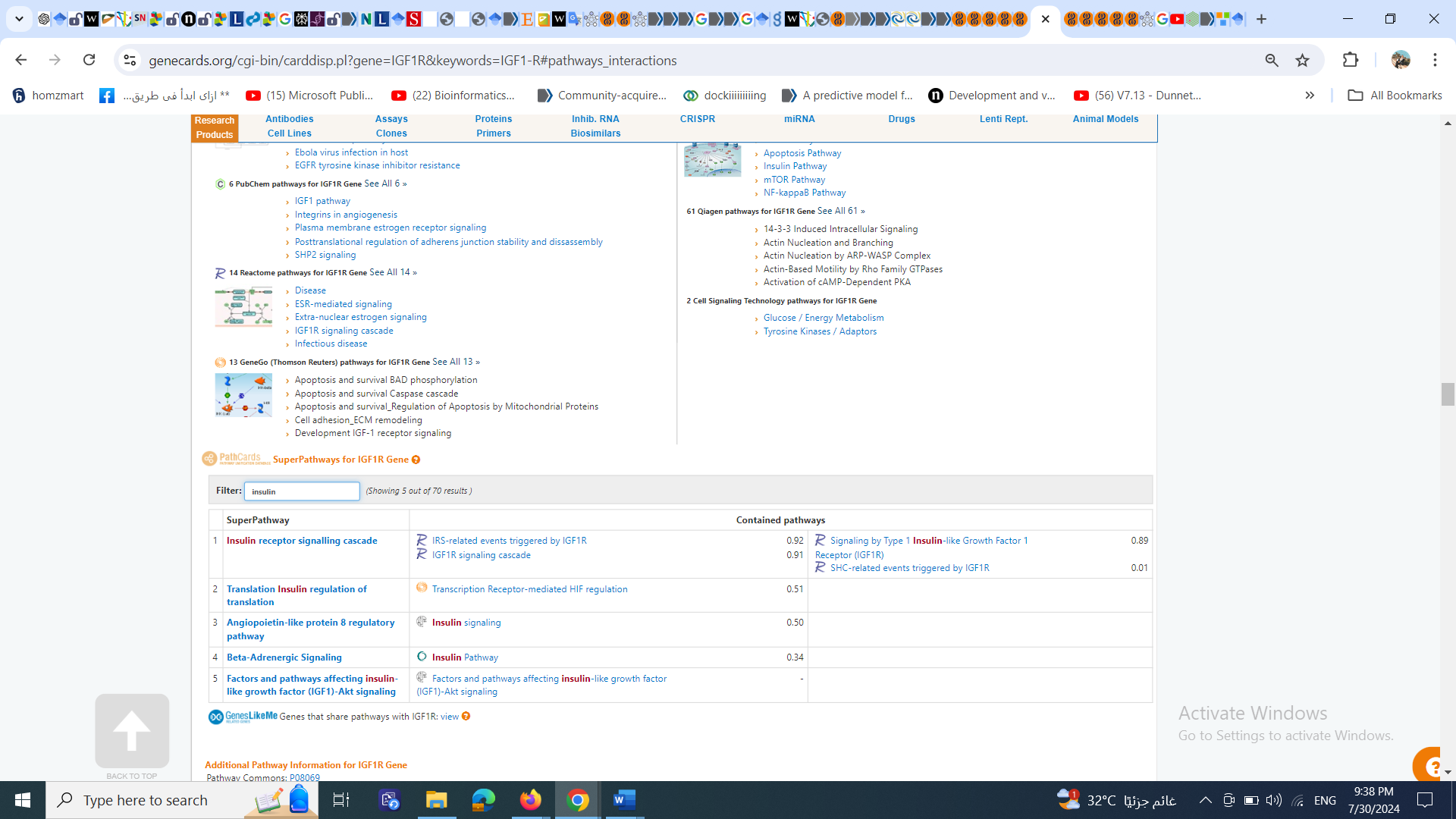


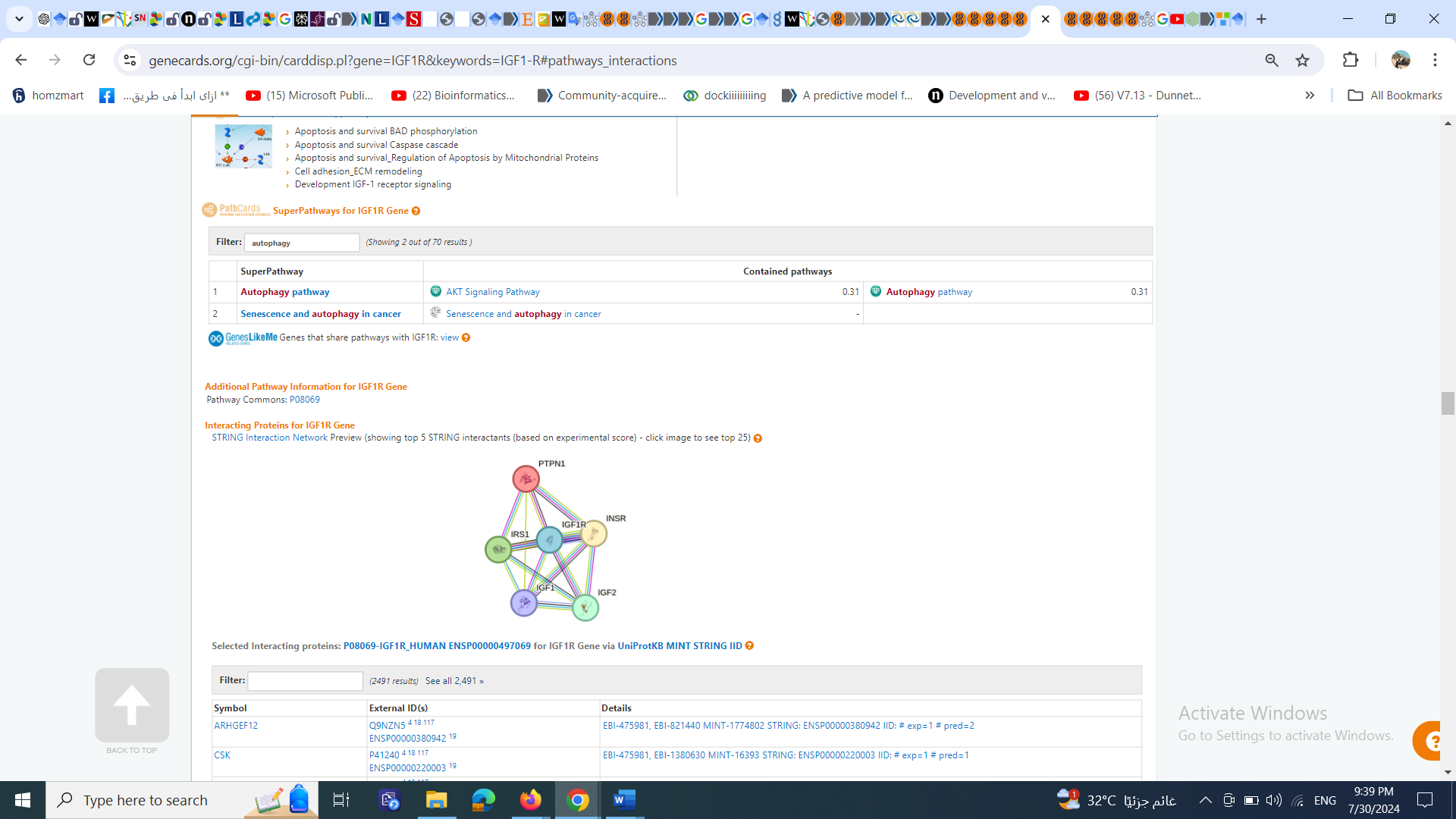


**HSPA1B**


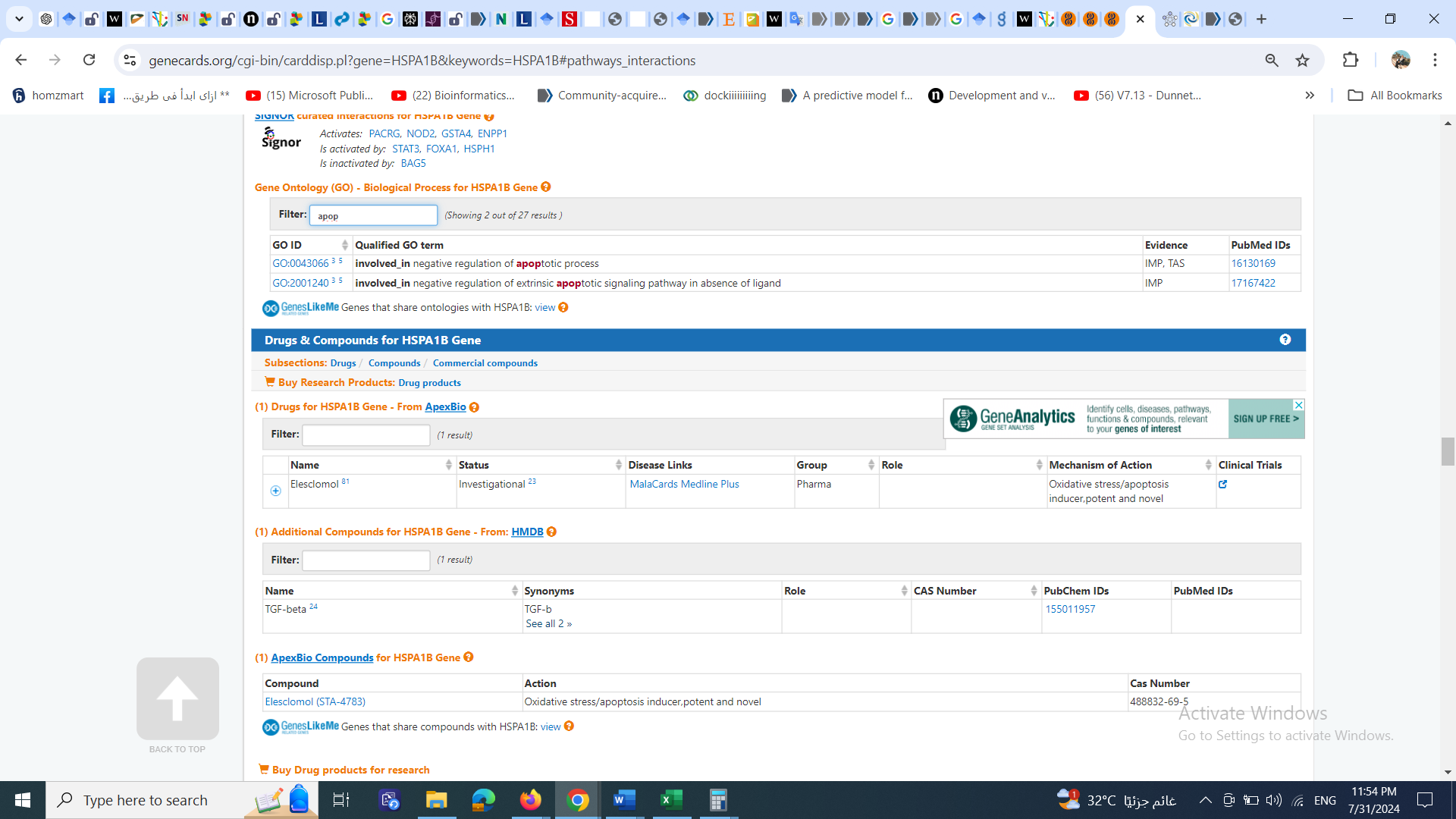


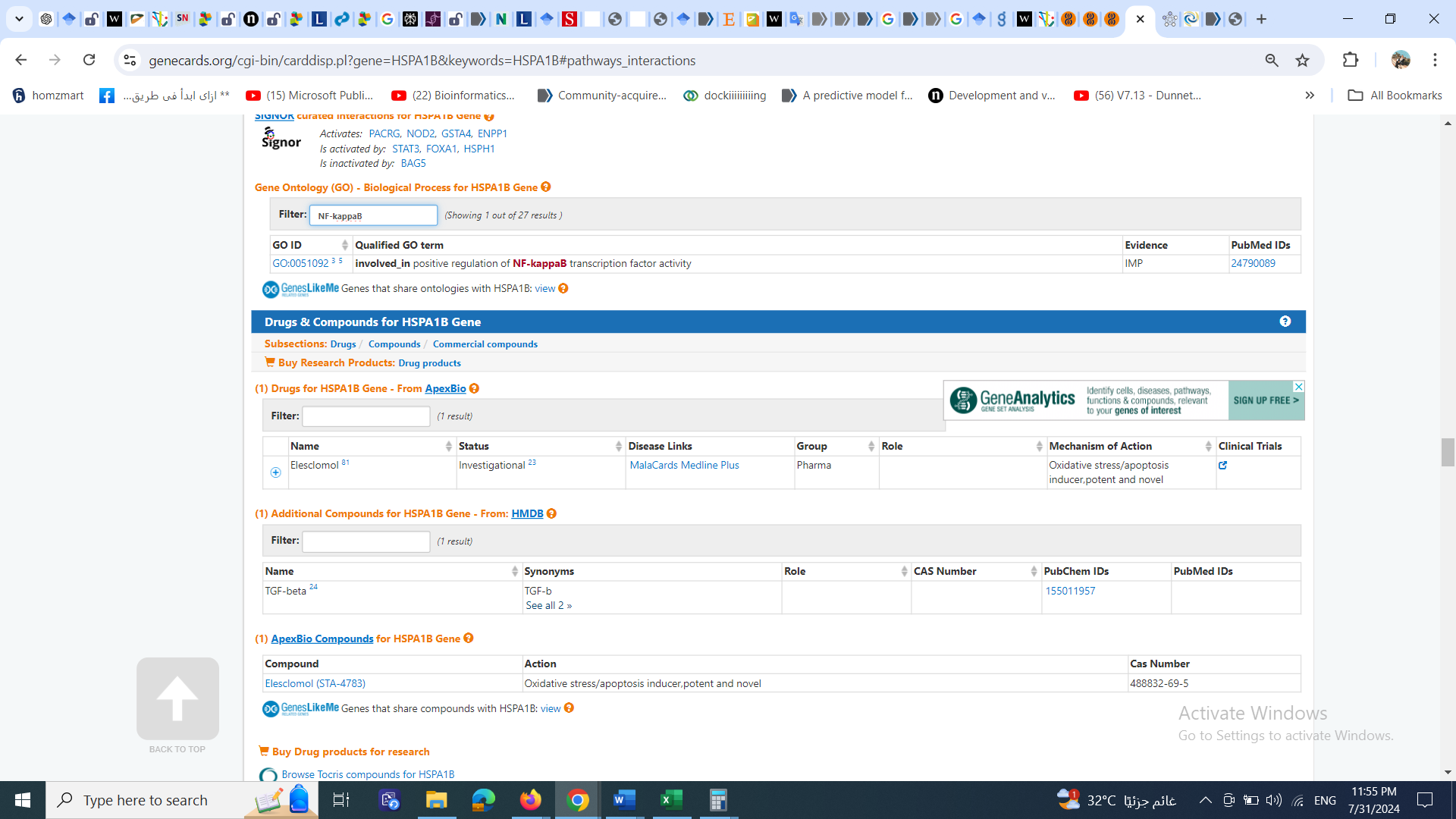


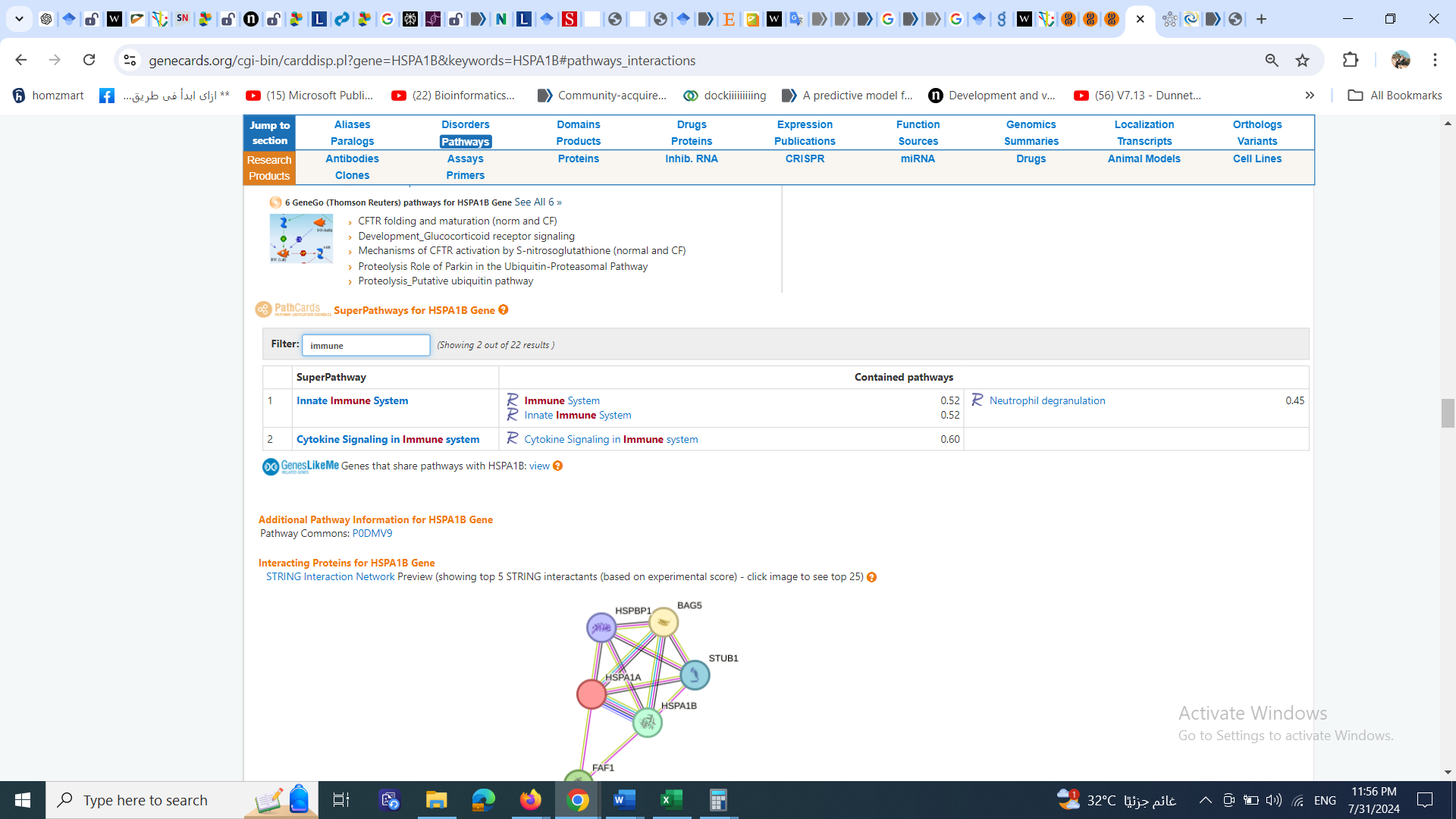


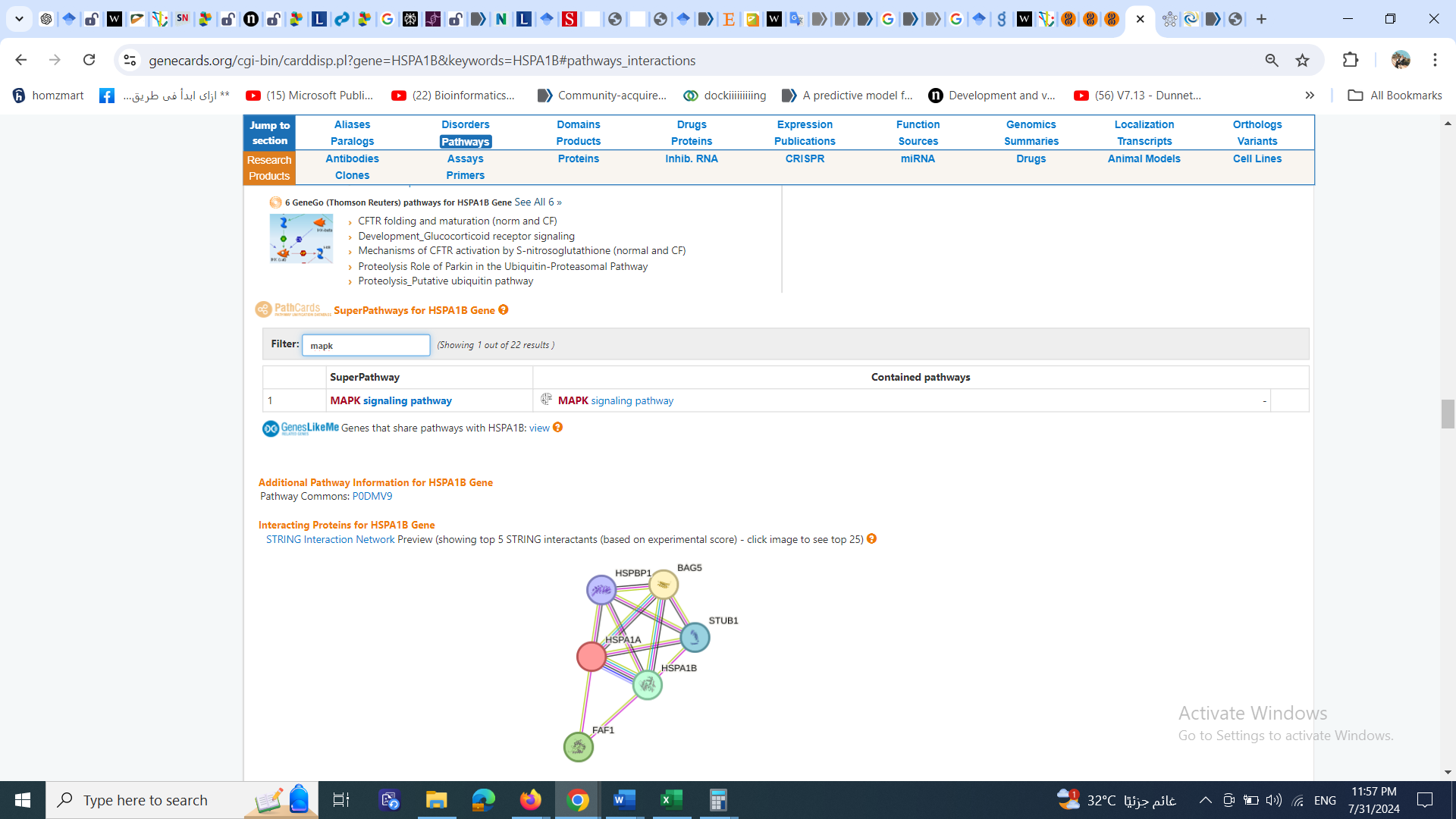


**RB1CC1**


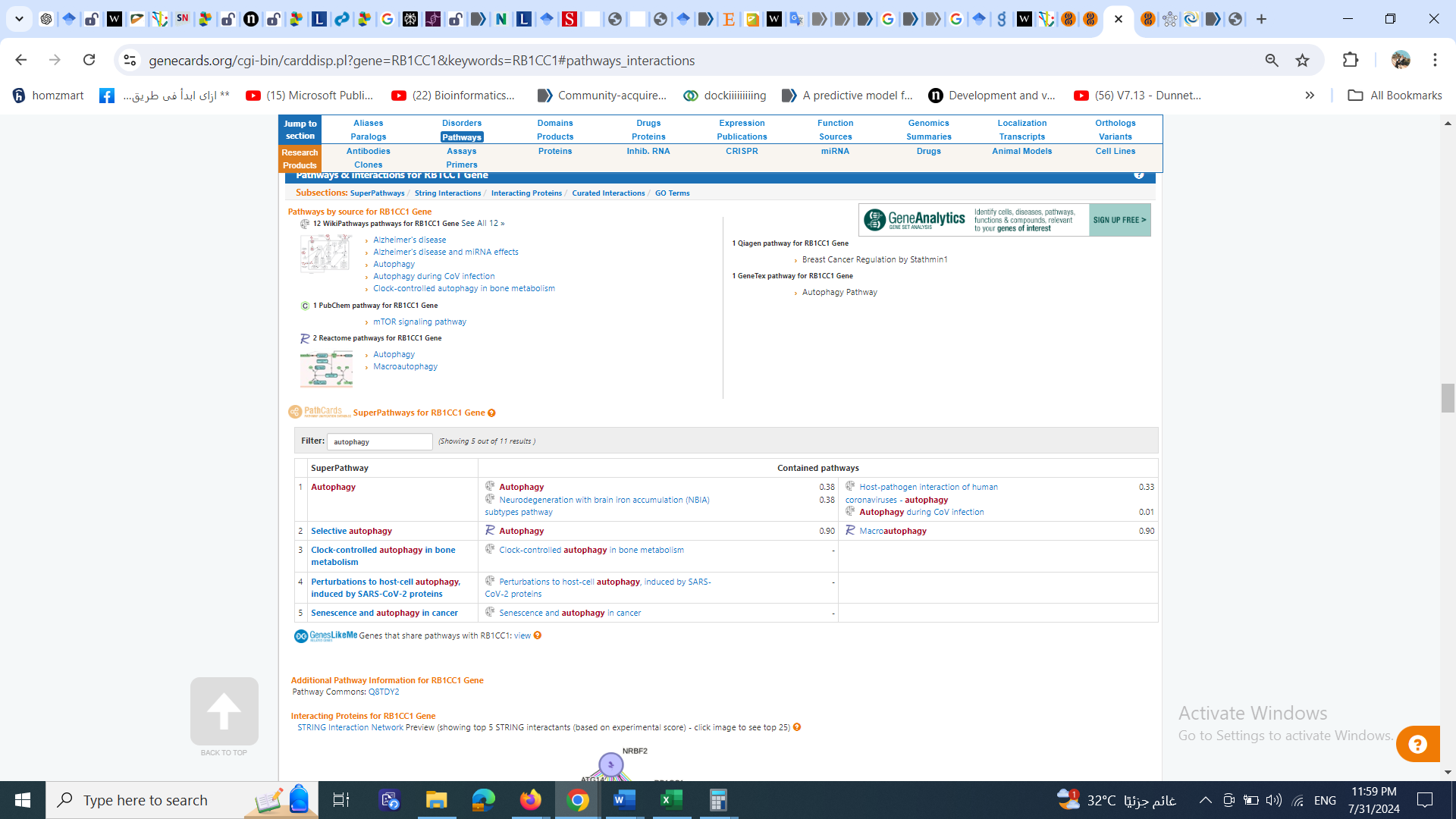


**RET**


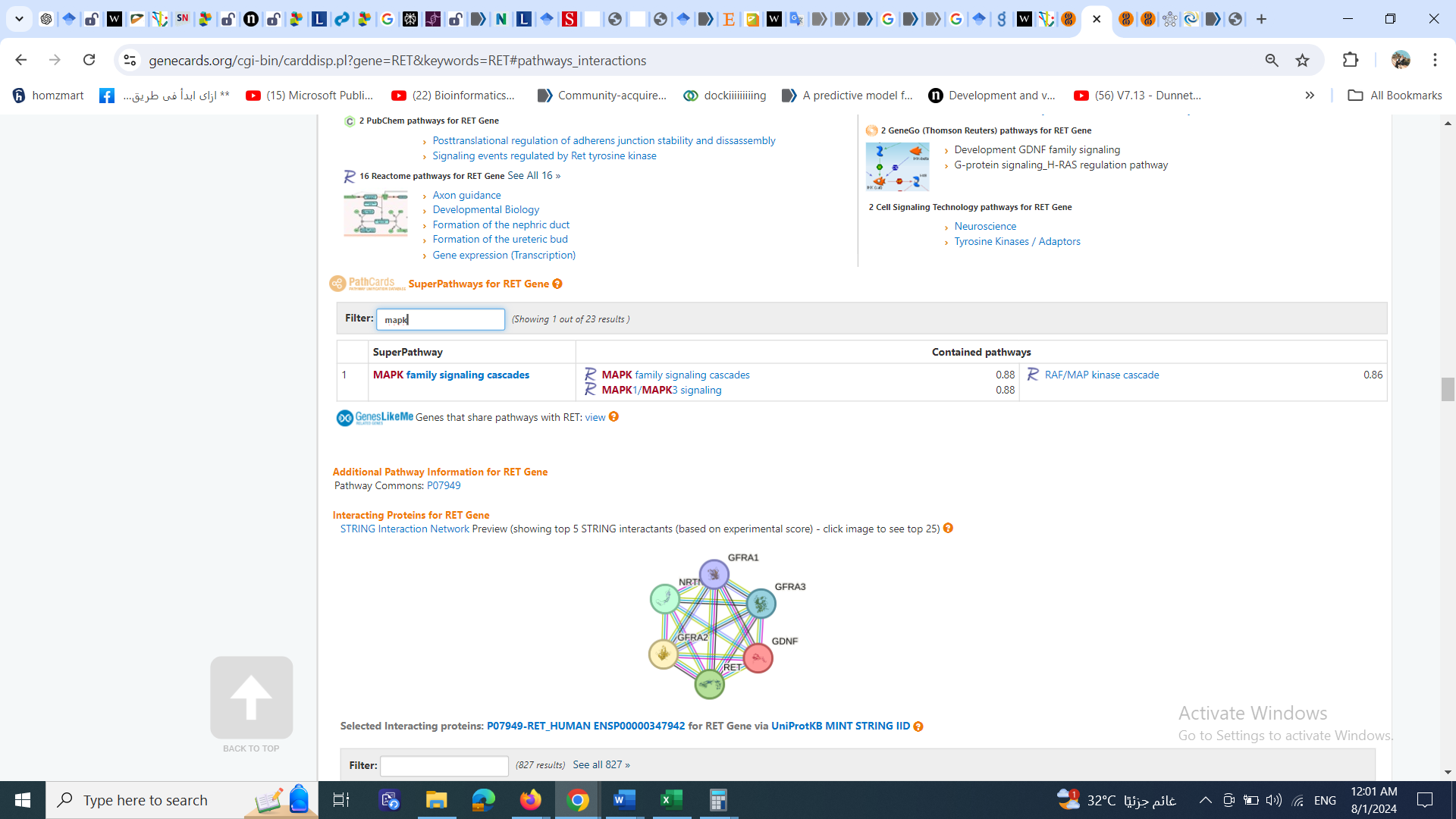


NFKB1


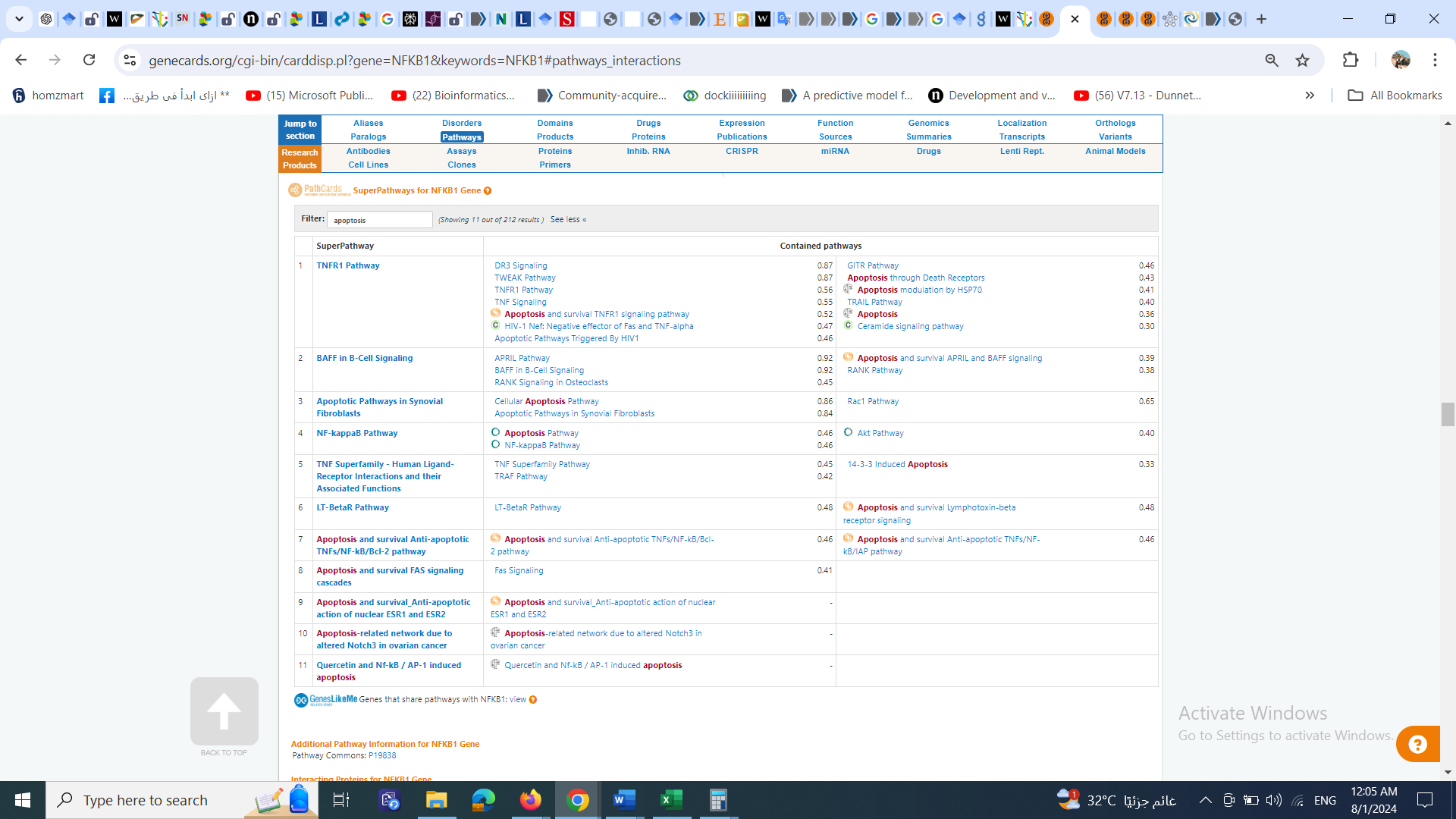


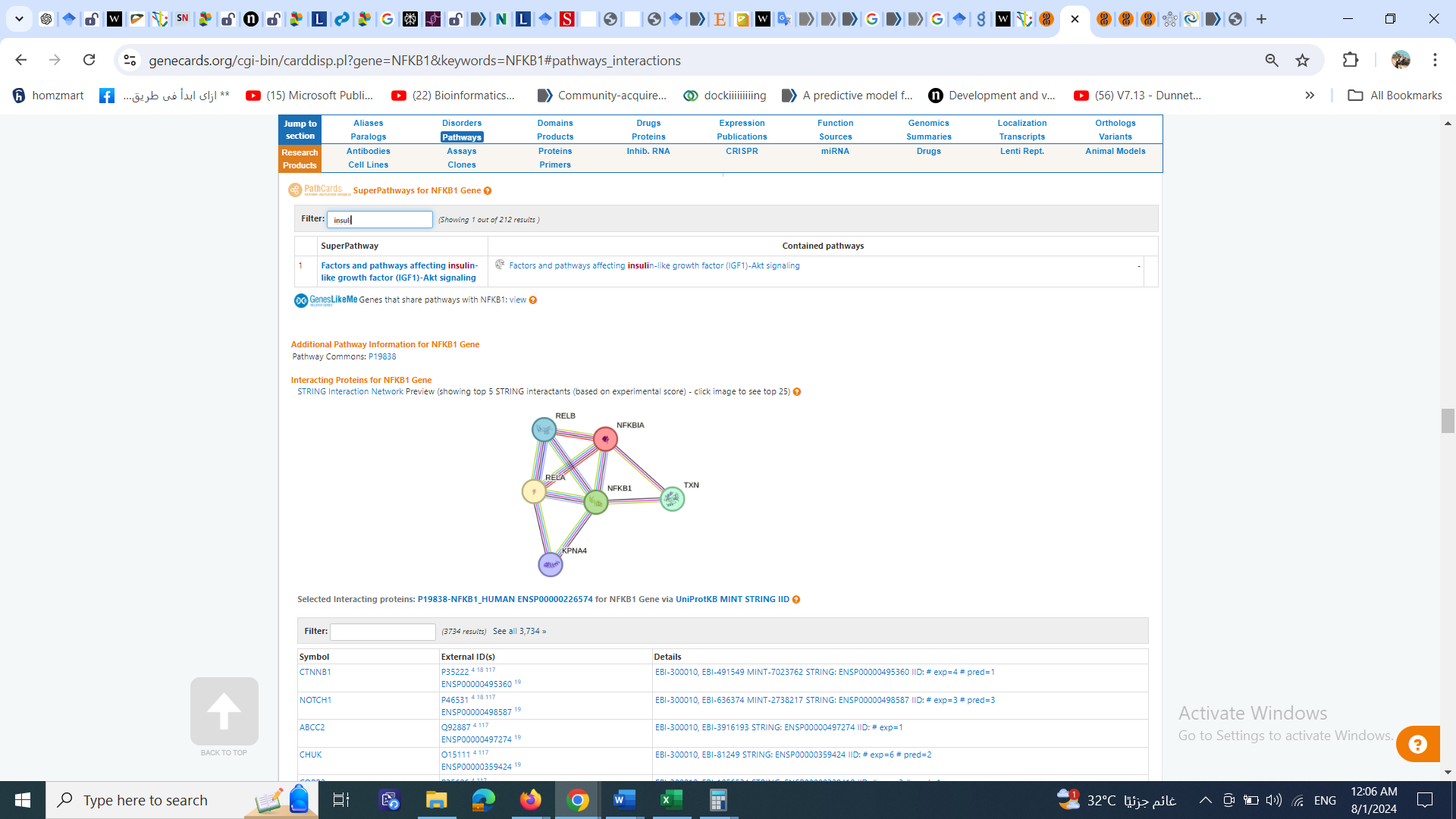


***MTOR***


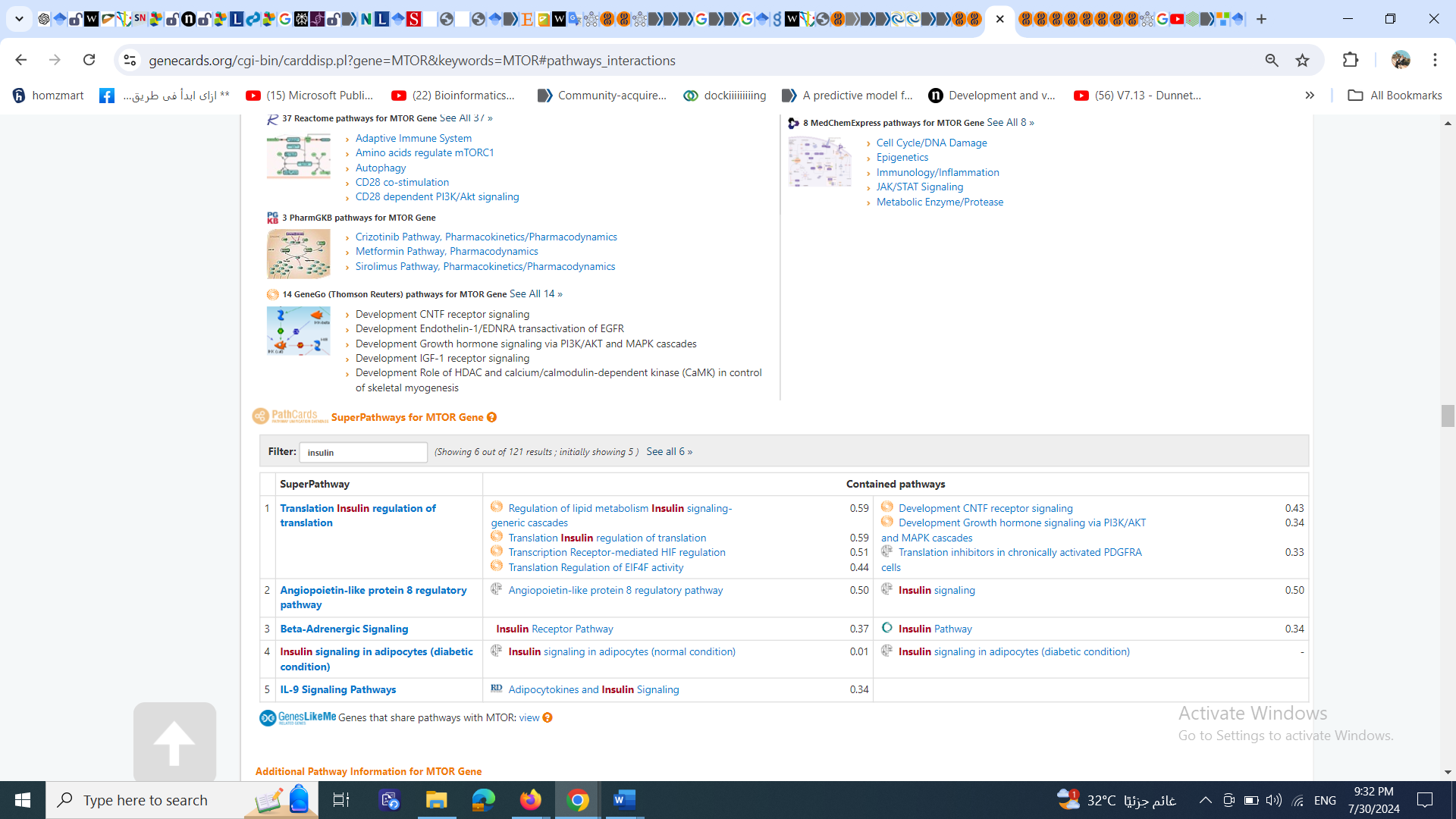


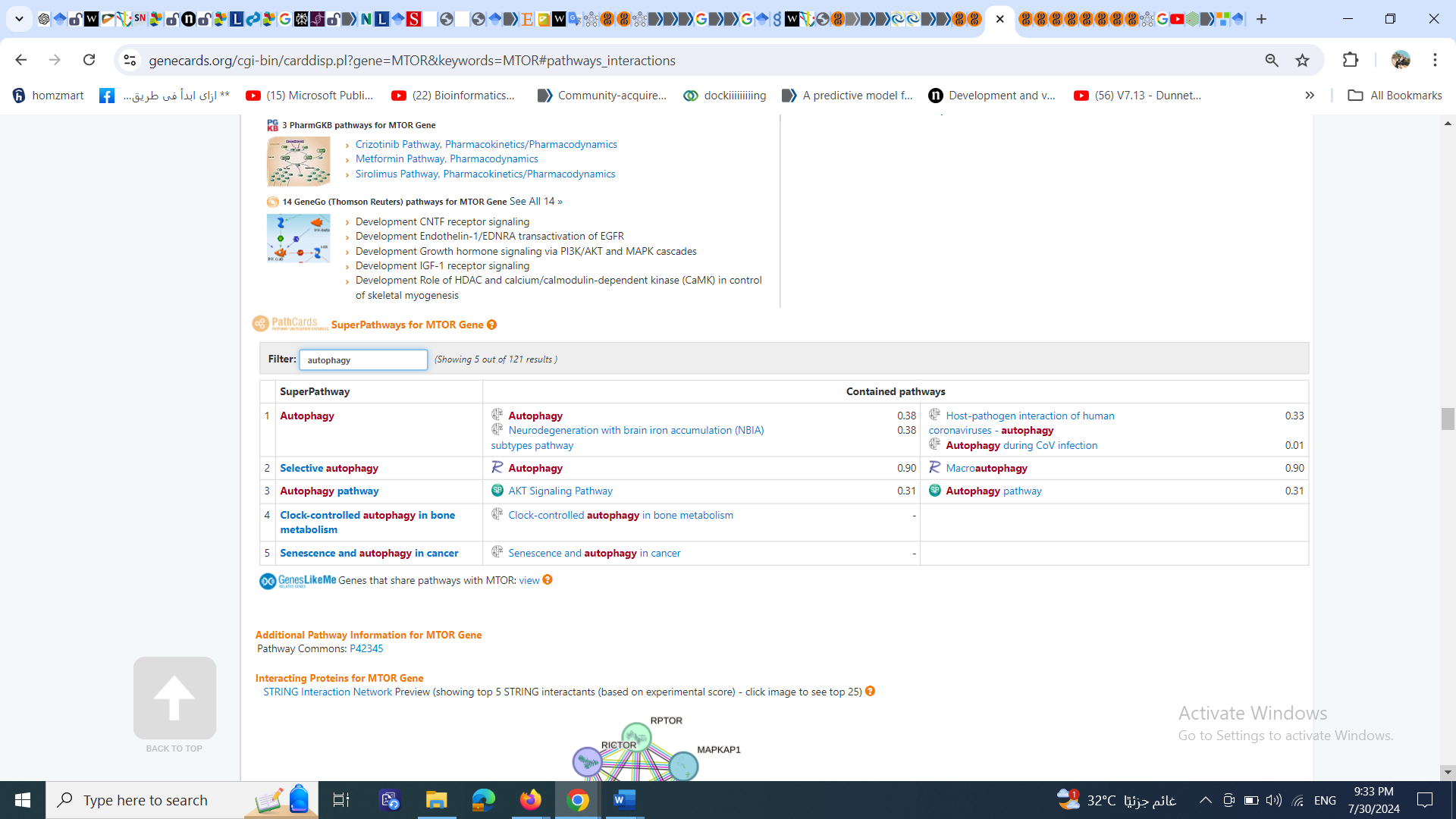


DDX58


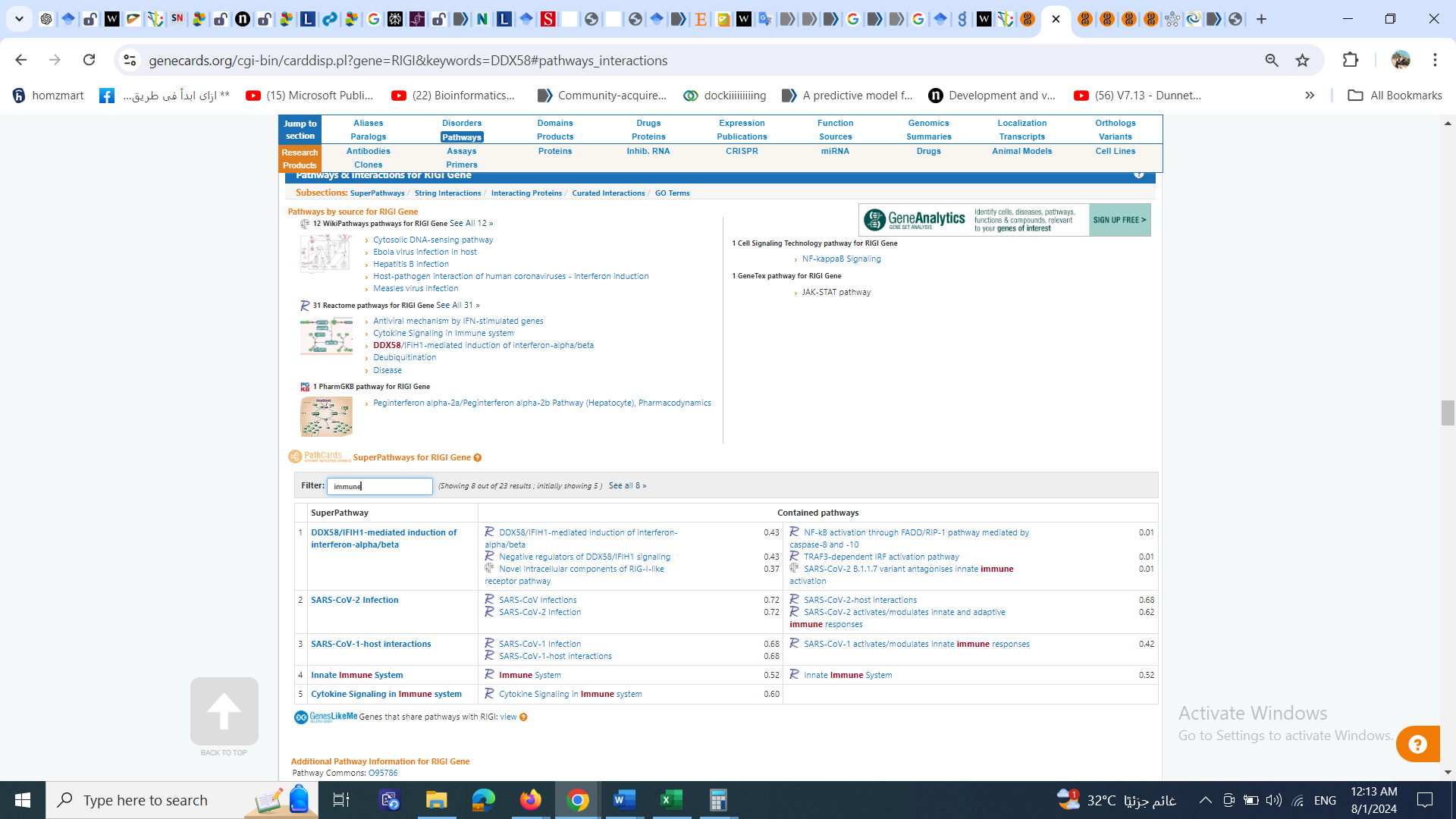


**Figure S2. Protein-Protein Interaction analysis of the retrieved mRNAs:**


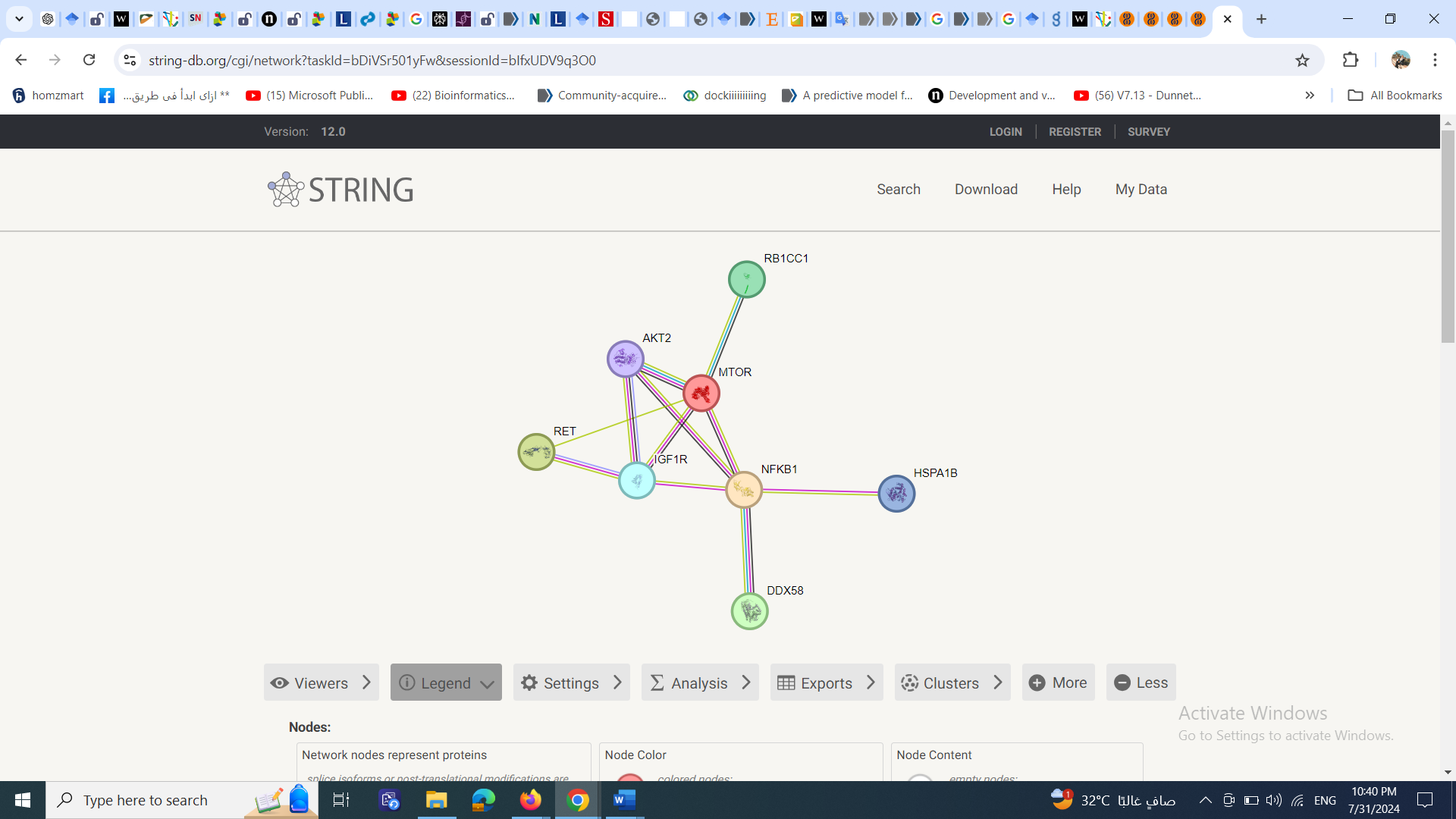


**Figure S3. Validation of the interaction between the selected mRNAs and the retrieved miRNAs from the mirWalk (**[**http://mirwalk.umm.uni-heidelberg.de/**](http://mirwalk.umm.uni-heidelberg.de/)**).**

**hsa-miR-15b**


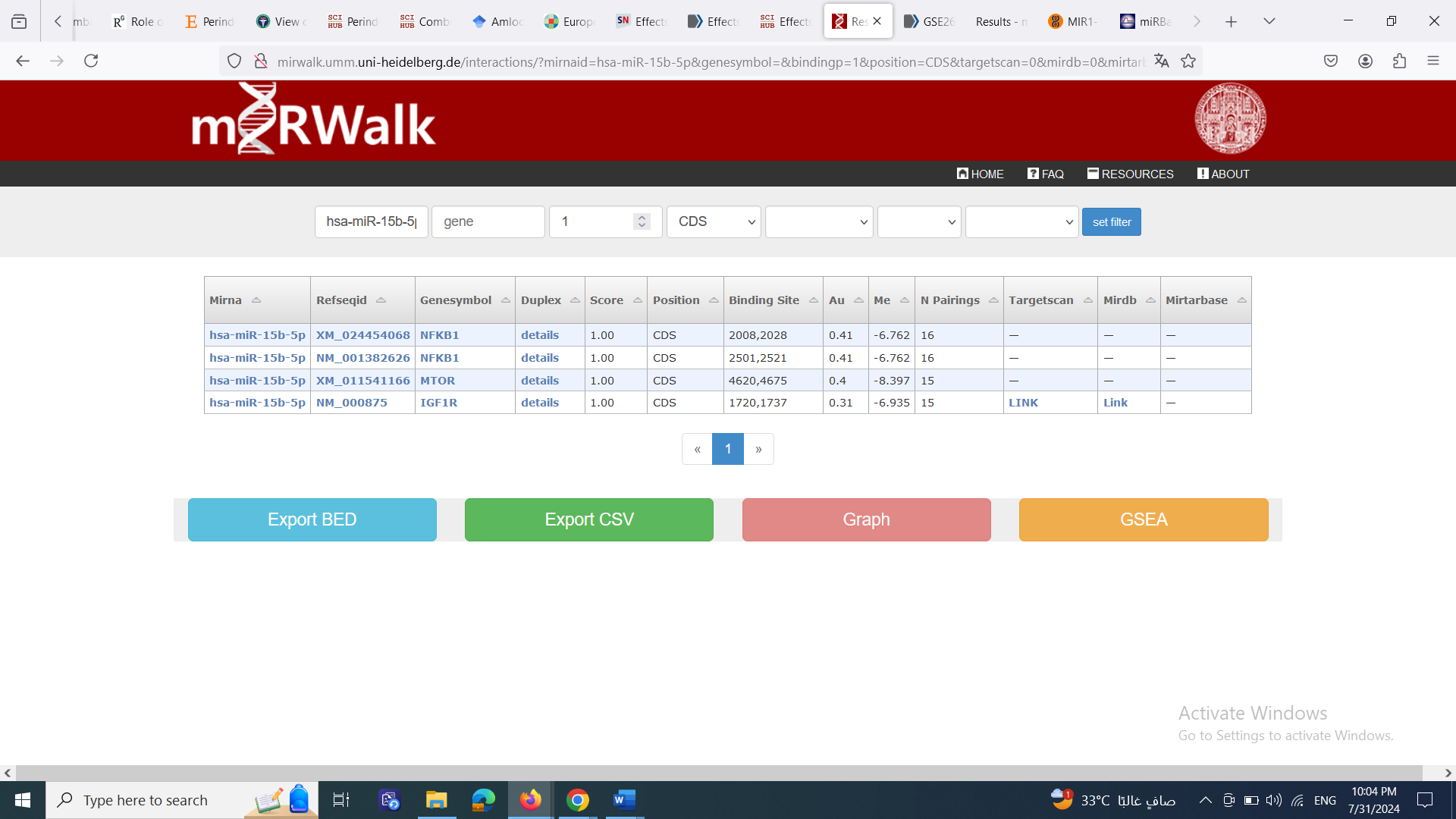


hsa-miR-3163


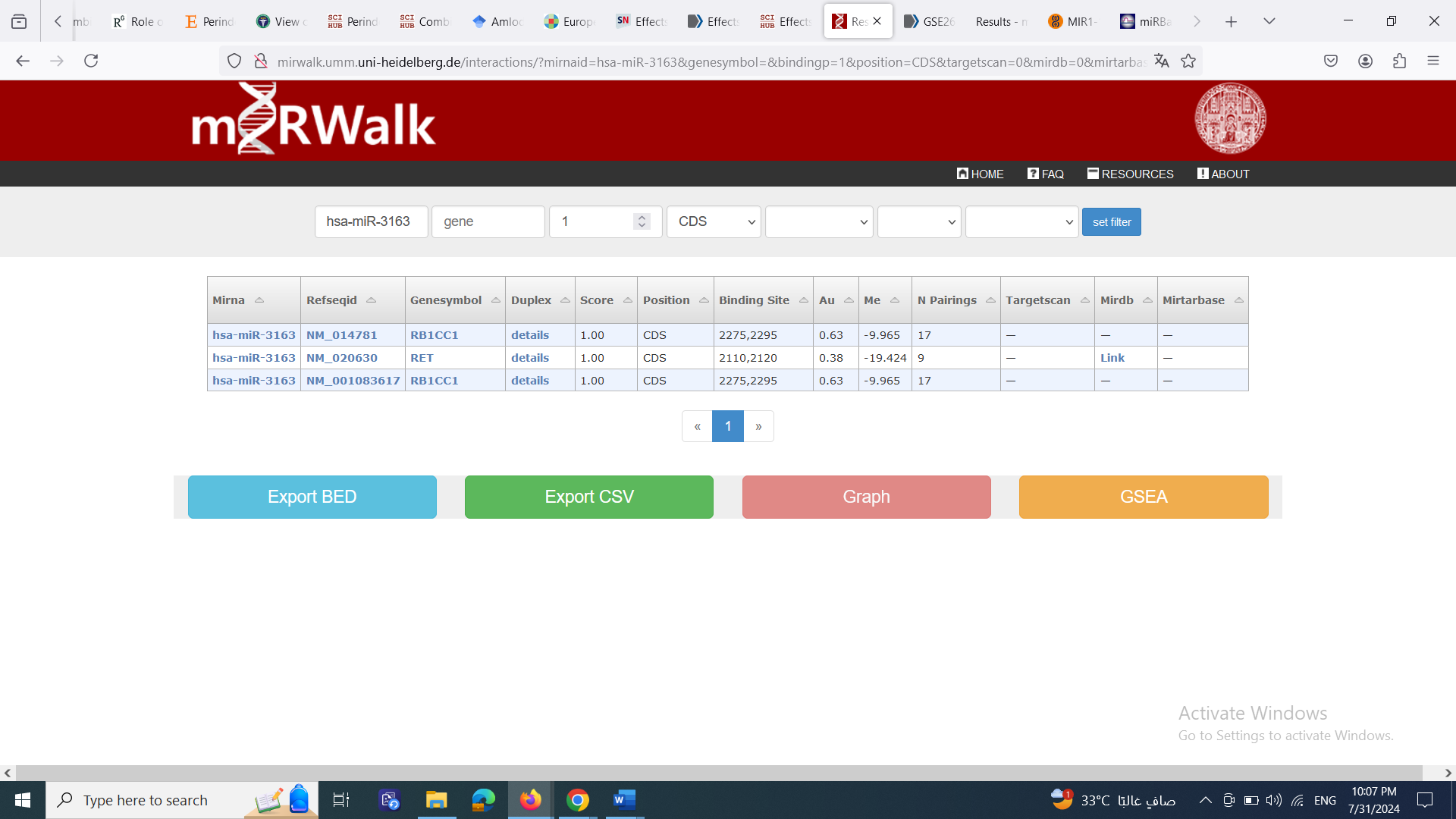


hsa-miR-611


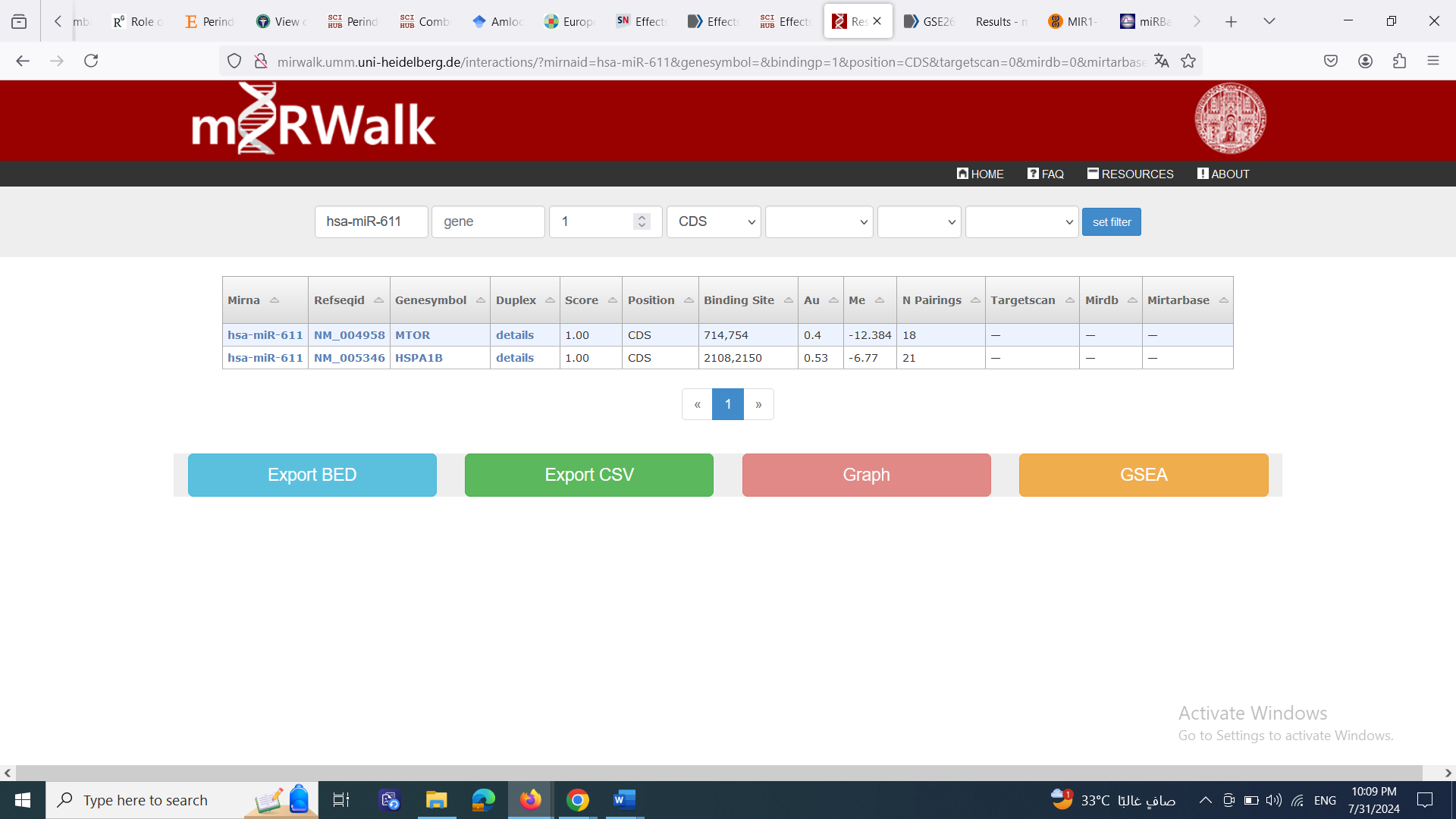


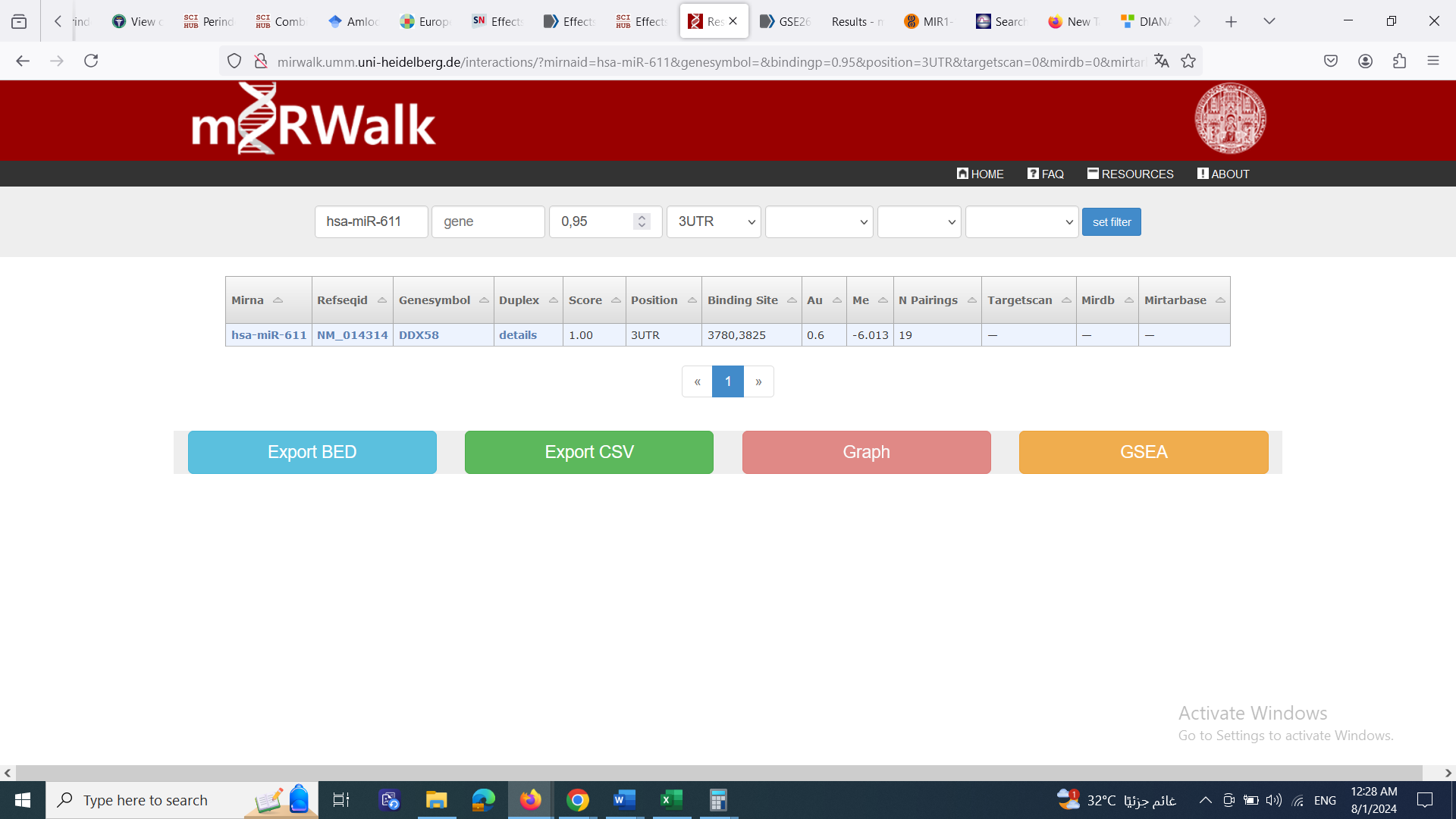


hsa-miR-30a


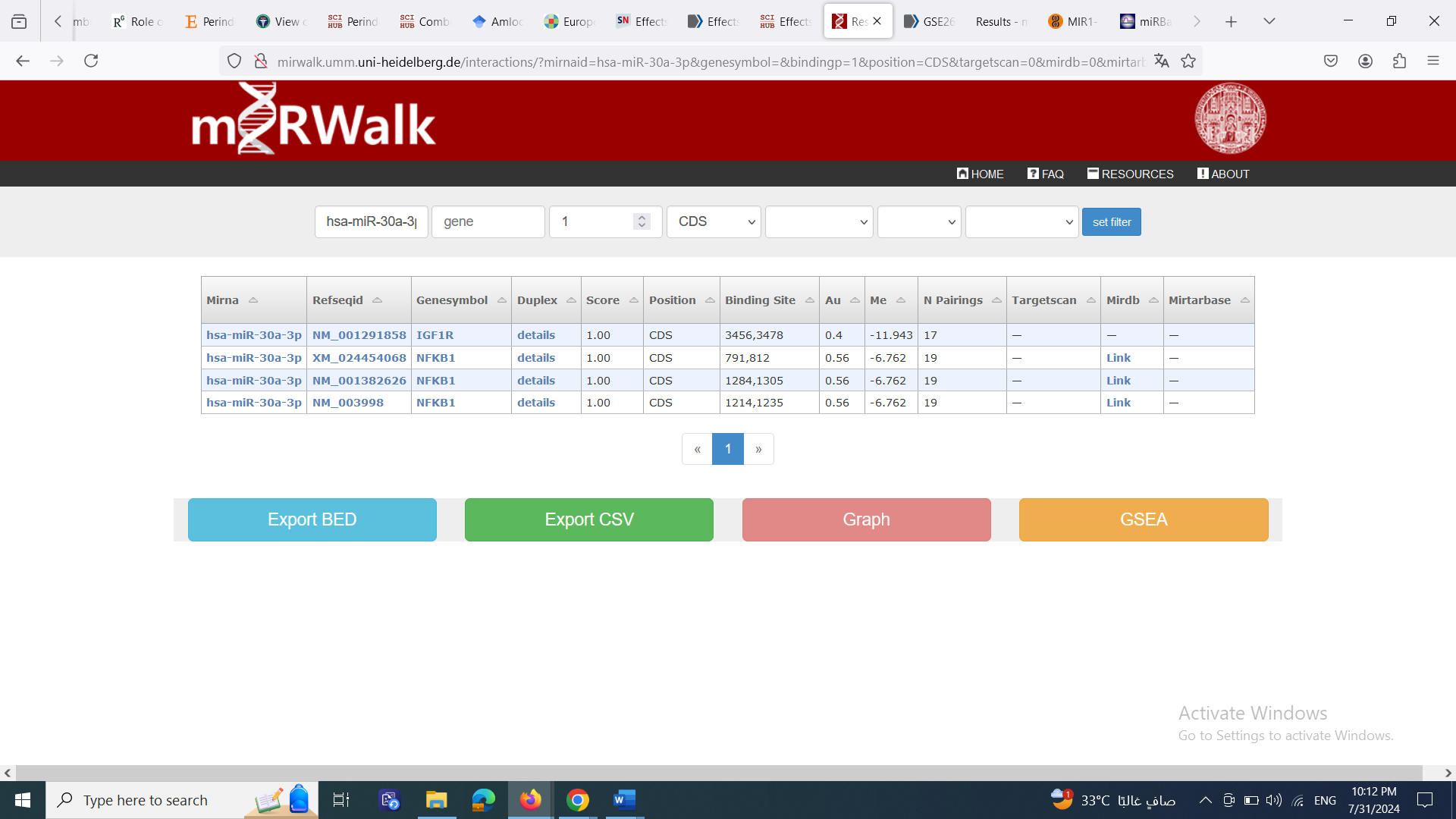


hsa-miR-342


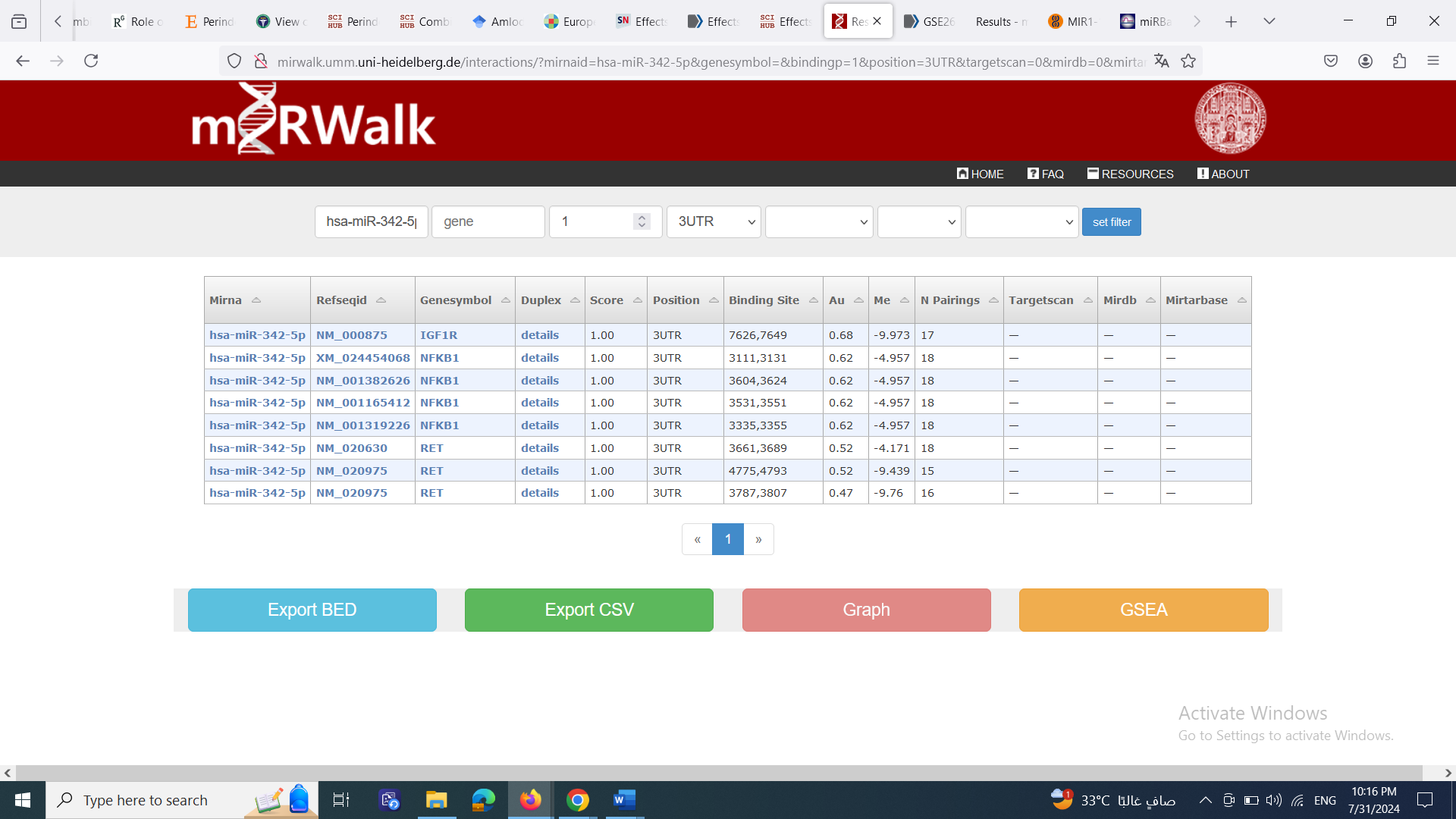


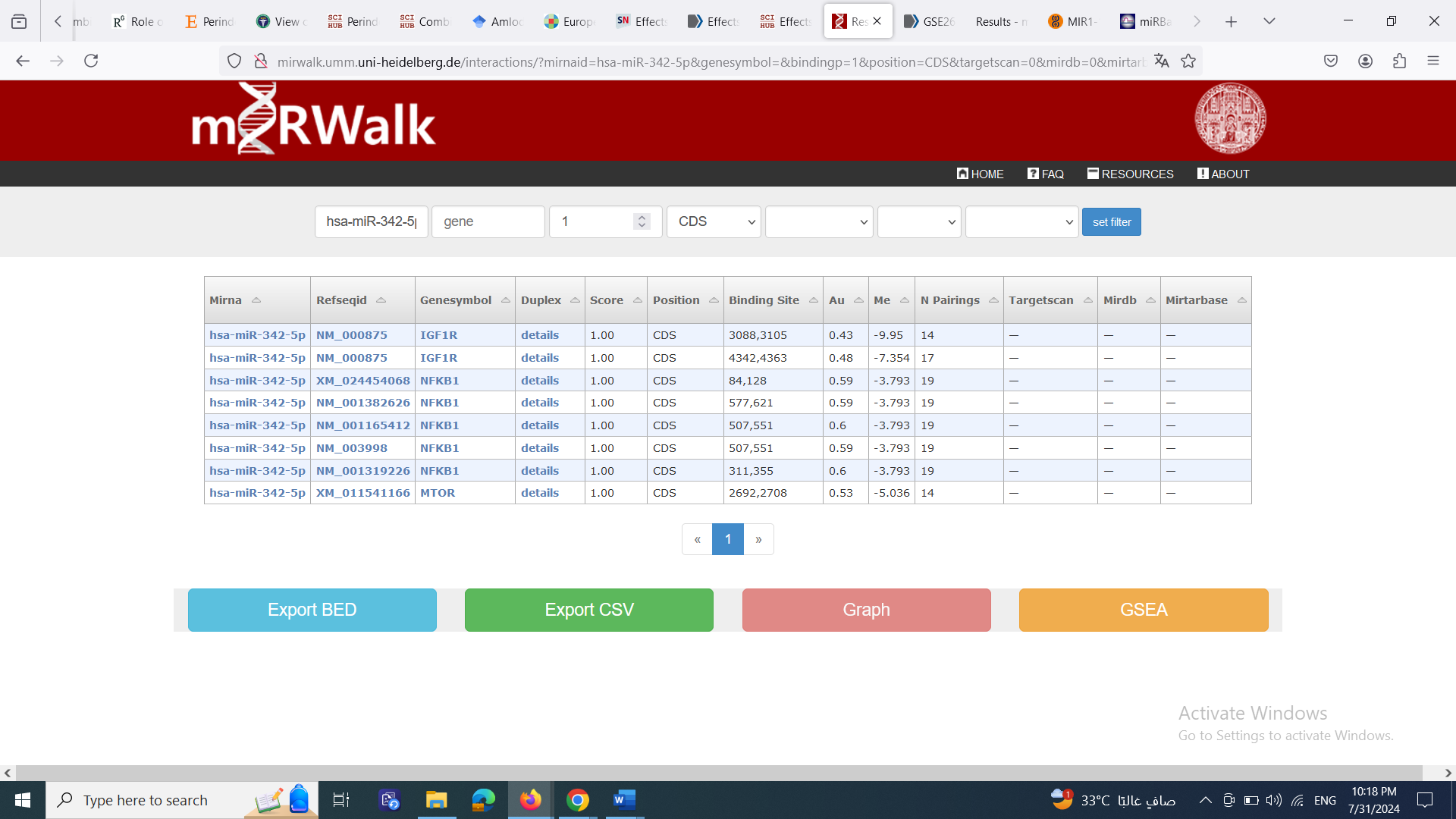


hsa-miR-636


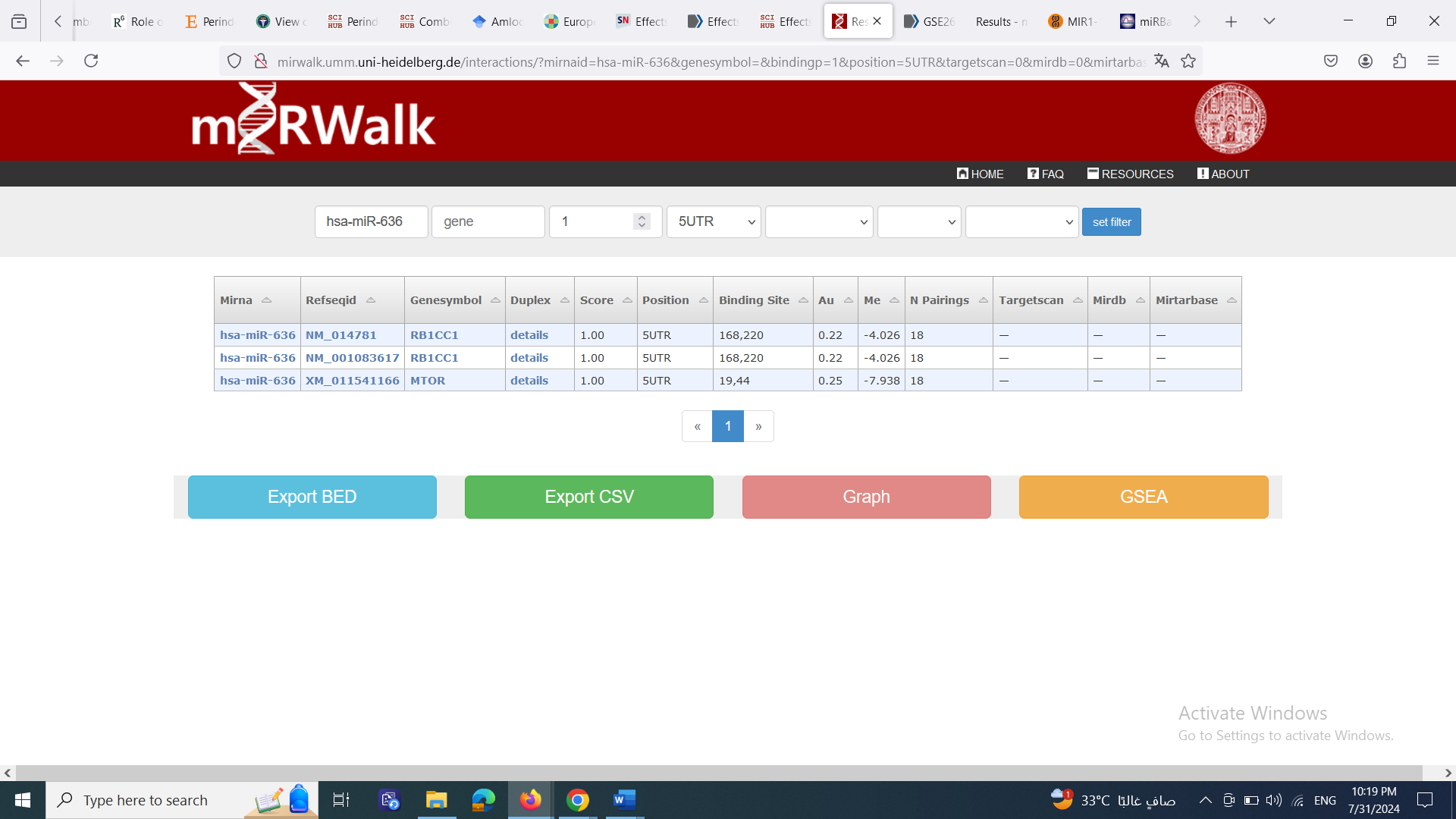


Table 2s: Epigenetic regulators of retrieved genes.

|  | NFKB1 | MTOR | IGF1R | RET | RB1CC1 | HSPA1B | DDX58 |
| --- | --- | --- | --- | --- | --- | --- | --- |
| miR-15b-5p | √ | √ | √ |  |  |  |  |
| miR-342-5p | √ | √ | √ | √ |  |  |  |
| miR-636 |  | √ |  |  | √ |  |  |
| miR-611 |  | √ |  |  |  | √ | √ |
